# Halogen Bond to Experimentally Significant N-Heterocyclic Carbenes (I, IMe_2_, I^*i*^Pr_2_, I^*t*^Bu_2_, IPh_2_, IMes_2_, IDipp_2_, IAd_2_; I = Imidazol-2-ylidene)

**DOI:** 10.3390/ijms24109057

**Published:** 2023-05-21

**Authors:** Mirosław Jabłoński

**Affiliations:** Faculty of Chemistry, Nicolaus Copernicus University in Toruń, Gagarina 7, 87-100 Torun, Poland; teojab@chem.umk.pl; Tel.: +48-056-611-4695

**Keywords:** carbene, N-heterocyclic carbene, NHC, halogen bond, XB, intermolecular interaction, steric effect, spatial hindrance

## Abstract

The subjects of the article are halogen bonds between either XCN or XCCH (X = Cl, Br, I) and the carbene carbon atom in imidazol-2-ylidene (I) or its derivatives (IR${}_{2}$) with experimentally significant and systematically increased R substituents at both nitrogen atoms: methyl = Me, *iso*-propyl = ${}^{i}$Pr, *tert*-butyl = ${}^{t}$Bu, phenyl = Ph, mesityl = Mes, 2,6-diisopropylphenyl = Dipp, 1-adamantyl = Ad. It is shown that the halogen bond strength increases in the order Cl < Br < I and the XCN molecule forms stronger complexes than XCCH. Of all the carbenes considered, IMes${}_{2}$ forms the strongest and also the shortest halogen bonds with an apogee for complex IMes${}_{2}\cdots$ICN for which $D_{0}$ = 18.71 kcal/mol and $d_{C\cdots I}$ = 2.541 Å. In many cases, IDipp${}_{2}$ forms as strong halogen bonds as IMes${}_{2}$. Quite the opposite, although characterized by the greatest nucleophilicity, I${}^{t}$Bu${}_{2}$ forms the weakest complexes (and the longest halogen bonds) if X ≠ Cl. While this finding can easily be attributed to the steric hindrance exerted by the highly branched *tert*-butyl groups, it appears that the presence of the four C-H⋯X hydrogen bonds may also be of importance here. Similar situation occurs in the case of complexes with IAd${}_{2}$.

## 1. Introduction

Carbenes [1,2,3,4,5,6,7,8,9,10], especially the N-heterocyclic (NHC) [11,12,13,14,15,16,17,18,19,20,21,22,23] ones, are of fundamental importance in organic and organometallic chemistry as well as in homogeneous catalysis [24,25,26,27,28,29,30,31,32,33,34,35], material chemistry [36,37], and medicine [38,39]. The imidazol-2-ylidene derivatives are especially important in this respect. The significant reactivity of these derivatives and other NHCs results from the presence of a lone electron pair on the carbene carbon atom, which makes these carbenes good nucleophilic reagents. In this respect, carbenes are most often considered electron charge donors, i.e., Lewis bases, in combination with transition metal atoms, usually forming strong complexes [40,41,42,43,44,45,46,47,48,49,50,51,52,53,54,55,56]. However, the carbene carbon atom readily forms contacts with atoms other than transition metals as well, forming hydrogen bonds [57,58,59,60,61,62,63,64,65], lithium bonds [66,67,68], beryllium bonds [69,70,71,72], magnesium bonds [73,74,75], triel bonds [76,77,78,79], tetrel bonds [80,81,82], pnictogen bonds [83,84,85], chalcogen bonds [86], halogen bonds [87,88,89,90,91,92,93,94,95], and even aerogen bonds [96]. However, it is worth mentioning here that in addition to the nucleophilic properties of carbenes resulting from the presence of a lone electron pair on the carbene carbon atom, singlet carbenes [97,98,99,100,101,102,103] also exhibit electrophilic properties [104,105,106,107,108,109] associated with the presence of an empty *p*-orbital perpendicular to the plane of the molecule. Although the target of electrophilic attack is most often nitrogen or phosphorus [105], it has recently been shown that the carbene carbon atom can also interact with the Si-H bond in silane to form a specific type of tetrel bond [107,108,109].

The subject of this article is a special case of the halogen bond, because the donor of the lone electron pair is the carbene carbon atom and not, as in most cases, a strongly electronegative atom such as, e.g., N, P, O, S, or another halogen [110,111,112,113]. Thus, the carbene molecule here is a Lewis base, while the molecule with the contacting halogen atom acting through the σ-hole [114,115,116,117,118] is a Lewis acid. The first adduct related to the interaction between the carbene carbon atom and the halogen atom was obtained by Arduengo III et al. [87] in 1991. It was formed by the reaction of 1,3-di-1-adamantyl-imidazol-2-ylidene (IAd${}_{2}$) with iodopentafluorobenzene. Subsequently, Kuhn et al. [88] obtained a stable carbene iodine adduct by reacting 1,3-diethyl-4,5-dimethylimidazol-2-ylidene with iodine. These two examples clearly show that iodine is the best halogen atom as an electron pair acceptor. This is, of course, due to the fact that the σ-hole is particularly well developed on the iodine atom. The first theoretical studies of the halogen bond involving a carbene molecule were made by Li et al. [89] only in 2009. Namely, they performed calculations for five complexes H${}_{2}$C⋯BrH, F${}_{2}$C⋯BrH, Me${}_{2}$C⋯BrH, H${}_{2}$C⋯BrCCH, and H${}_{2}$C⋯BrCN. It was not until 2013 that Esrafili and Mohammadirad studied the H${}_{2}$C⋯XCCY (X = Cl, Br, I; Y = H, F, COF, COOH, CF${}_{3}$, NO${}_{2}$, CN, NH${}_{2}$, CH${}_{3}$, OH) complexes [90] and the H${}_{2}$C⋯XCN⋯XCN linear trimers (X = F, Cl, Br, I) [91]. As can be seen, the first three computational works concerned the simplest carbene CH${}_{2}$, i.e., methylene, or its simple derivatives. Only in 2014, Donoso-Tauda et al. [92] performed calculations for complexes involving 1,3-dimethylimidazole-2-ylidene (IMe${}_{2}$). The donors of the X halogen atom (X = Cl, Br) were X-A molecules, where A = F, Cl, Br, CN, CCH, CF${}_{3}$, CH${}_{3}$ or H. Dimers between XCN (X = Cl, Br, I) and imidazol-2-ylidene derivatives, including, e.g., 1,3-di-*tert*-butyl-imidazole-2-ylidene (I${}^{t}$Bu${}_{2}$) and 1,3-diphenylimidazole-2-ylidene (IPh${}_{2}$) were studied in the same year by Lv et al. [93]. Then, Del Bene et al. [94] performed theoretical studies of the halogen bond between ClCCH, ClCN, ClNC, or ClF and some rather exotic small carbenes. Quite recently, Grabowski performed calculations for imidazol-2-ylidene complexes with XCCH, XCN, and X${}_{2}$ (X = F, Cl, Br, I) [95]. As might be expected, the F molecules did not form a halogen bond to the carbene carbon atom. In addition to the aforementioned work on halogen bonds involving carbenes, it is also worth mentioning the recent theoretical article by Sanyal and Esterhuysen [119] on halogen bonds involving carbones and the hot paper on halogen complexes of anionic NHCs by Frosch et al. [120].

**Figure 1:**
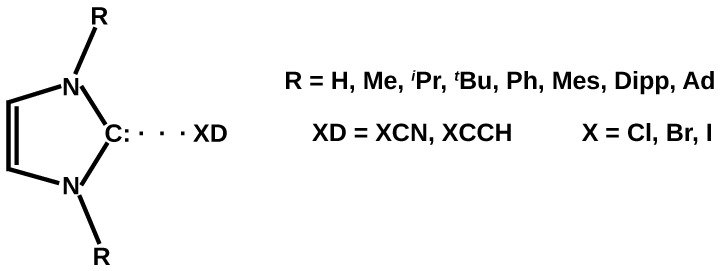
General scheme of the IR${}_{2}\cdots$XD complexes (R = H, Me, ${}^{i}$Pr,${}^{t}$Bu, Ph, Mes, Dipp, Ad; XD = XCN, XCCH; X = Cl, Br, I). The colon on the carbene carbon atom represents a lone electron pair.

The examples cited [89,90,91,92,93,94,95] clearly show that currently there is practically no theoretical work on halogen bonding involving larger and experimentally significant NHC-type carbenes. In the vast majority of cases, these are derivatives of imidazol-2-ylidene containing large groups at both nitrogens of the heterocycle, e.g., 1-adamantyl (= Ad), 2,6-diisopropylphenyl (= Dipp), mesityl (= Mes), *tert*-butyl (= ${}^{t}$Bu), or *iso*-propyl (= ${}^{i}$Pr). It is enough to recall here that the first adduct of the carbene and halogen compound was a derivative of 1,3-di-1-adamantyl-imidazol-2-ylidene (IAd${}_{2}$) [87]. Unfortunately, calculations with such large substituents are computationally very expensive. Nevertheless, the aim of this article is to systematically investigate the characteristics of the halogen bond between the most popular halogen donors XCCH and XCN (X = Cl, Br, I) and the carbene carbon atom of the experimentally significant imidazol-2-ylidene derivatives. Additionally, imidazol-2-ylidene itself and its several smaller derivatives are also investigated. Thus, the considered carbenes are imidazol-2-ylidene (I) and its derivatives (IR${}_{2}$) with progressively larger substituents (R) at the heterocyclic nitrogens: methyl = Me, *iso*-propyl = ${}^{i}$Pr, *tert*-butyl = ${}^{t}$Bu, phenyl = Ph, mesityl = Mes, 2,6-diisopropylphenyl = Dipp, 1-adamantyl = Ad. The general form of the considered IR${}_{2}\cdots$XD complexes is shown in Figure 1. Of particular interest is the structure of the obtained complexes IR${}_{2}\cdots$XCN and IR${}_{2}\cdots$XCCH, as well as the characteristics of the halogen bonds that hold them together.

## 2. Results and Discussion

### 2.1. Nucleophilicity of Carbenes

Before discussing the results of the analyzed complexes, it is worth first quantifying the nucleophilicity index, *N*, of the individual carbenes, as nucleophilicity measures the ability of a nucleophile to react at an electron-deficient center [121] and therefore will be useful in the initial estimation of the tendency of a given carbene to form a halogen bond. Unfortunately, in the literature, one can find several expressions for the nucleophilicity index. Therefore, four methods were used to determine it (Method I-IV, see the Methodology section) [122]. The obtained values are presented in Table 1.

The nucleophilicity index values obtained suggest that I (i.e., imidazol-2-ylidene) should have the lowest tendency to form a halogen bond, while I${}^{t}$Bu${}_{2}$ and IDipp${}_{2}$, on the contrary, should have the highest. It is worth noting that the nucleophilicity index clearly increased with the increase of the aliphatic substituent, e.g., in the case of Method III (it has been shown [122] that this method is superior to Method IV and especially I and II) we have: I (2.54) < IMe${}_{2}$ (2.67) < I${}^{i}$Pr${}_{2}$ (2.74) < I${}^{t}$Bu${}_{2}$ (2.84). Similarly, the insertion of three methyl groups or two *iso*-propyl groups into the side phenyl group also led to an increase in the nucleophilicity index value: I (2.54) < IPh${}_{2}$ (2.67) < IMes${}_{2}$ (2.75) < IDipp${}_{2}$ (2.89). Most likely, these trends can be explained by the increase in the inductive effect (+I). However, the values of the obtained nucleophilicity indices did not fully transform into the strength of the obtained halogen bonds (vide infra).

### 2.2. Characteristics of IR${}_{2}\cdots$XCCN and IR${}_{2}\cdots$XCN Dimers

Undoubtedly, the most important parameters describing halogen bonds in the IR${}_{2}\cdots$XCCH, and IR${}_{2}\cdots$XCN (X = Cl, Br, I) complexes are the dissociation energy ($D_{0}$), the halogen bond length ($d_{C\cdots X}$), and the C-X-C angle ($\alpha_{\mathrm{CXC}}$). The values of these parameters are shown in Table 2. In addition, this table also contains the values of the most important QTAIM [123] quantities: the electron density ($\rho_{C\cdots X}$) and its Laplasian ($\nabla^{2}\rho_{C\cdots X}$) and the total electronic energy density ($H_{C\cdots X}$) determined at the bond critical point of the C⋯X halogen bond.

As expected for a given X-donor molecule, the halogen bond strength increases in the order of Cl < Br < I. For example, for the simplest I⋯XCN complexes, the $D_{0}$ values are 5.87, 8.58, and 14.31 kcal/mol, respectively, and for the IDipp${}_{2}\cdots$XCN complexes, the values are 8.59, 10.95, and 17.56 kcal/mol. It is also seen that XCN molecules form stronger complexes with carbenes than XCCH, which proves that XCN are much better halogen donors.

**Figure 2:**
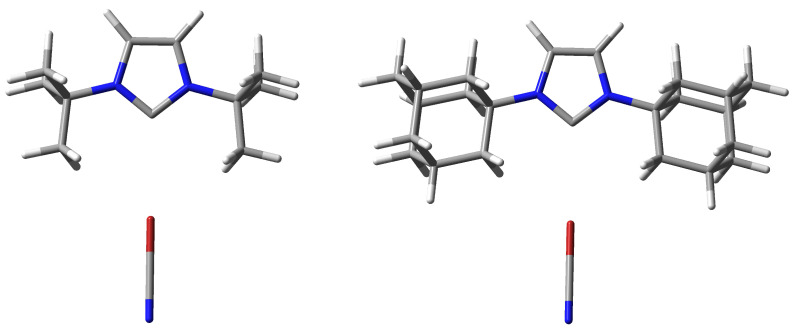
Structures of the I${}^{t}$Bu${}_{2}\cdots$BrCN and IAd${}_{2}\cdots$BrCN complexes.

However, it is more interesting to consider the strength of the complexes depending on the type of IR${}_{2}$ carbene molecule. As shown in the previous subsection (Table 1), the nucleophilicity index values determined suggest that unsubstituted imidazol-2-ylidene should form the weakest halogen bonds, while I${}^{t}$Bu${}_{2}$ and IDipp${}_{2}$ the strongest. Therefore, the $D_{0}$ values shown in Table 2 may seem surprising at first. Namely, although in the case of the halogen donors ClCN and ClCCH, imidazol-2-ylidene (I) indeed forms the weakest complexes (5.87 and 3.43 kcal/mol, respectively), in the case of the other halogen donors, the carbene I${}^{t}$Bu${}_{2}$ gives the weakest and not the strongest complexes (e.g., in the case of I${}^{t}$Bu${}_{2}\cdots$BrCCH the $D_{0}$ value is only 4.70 kcal/mol while for I⋯BrCCH it is 5.30 kcal/mol and for IAd${}_{2}\cdots$BrCCH it amounts to 5.32 kcal/mol). Moreover, the halogen bond length ($d_{C\cdots X}$) values presented in Table 2 clearly show that I${}^{t}$Bu${}_{2}$ forms the longest halogen bond in most cases, with XCN over 3.14 Å and with XCCH over 3.28 Å. At the same time, the halogen bonds formed by this carbene are always linear. What is more, the halogen bonds formed by I${}^{t}$Bu${}_{2}$ (as well as IAd${}_{2}$) are characterized by the smallest values of the electron density at the C⋯X bond critical point. For example, in the I${}^{t}$Bu${}_{2}\cdots$BrCCH and IAd${}_{2}\cdots$BrCCH complexes, $\rho_{C\cdots X}$ amounts to 0.010 a.u., whereas 0.017 a.u. in I⋯BrCCH. This is even more evident in systems with iodine, e.g., the $\rho_{C\cdots X}$ value in I${}^{t}$Bu${}_{2}\cdots$ICN and IAd${}_{2}\cdots$ICN complexes is 0.017 a.u., while, e.g., in the I⋯ICN complex, it is as much as 0.044 a.u. Thus, apparently, in the case of I${}^{t}$Bu${}_{2}$, the greatest length of halogen bonds (if X ≠ Cl) and their relatively weakest strength result from the large size of highly branched *tert*-butyl groups, which significantly impede the access of XCN and XCCH molecules to the lone electron pair on the carbene carbon atom. The similarly long halogen bonds obtained in the case of IAd${}_{2}$ (ca. 3.14 Å for XCN and over 3.28 Å for XCCH) most likely have the same reason, which is the significant spatial size of the Ad group (however, for the same X-donor, the halogen bond in the IAd${}_{2}\cdots$XD complex is about 0.5–0.8 kcal/mol stronger than in I${}^{t}$Bu${}_{2}\cdots$XD). The complex structures of I${}^{t}$Bu${}_{2}\cdots$BrCN and IAd${}_{2}\cdots$BrCN shown in Figure 2 represent a case of relatively long and weak halogen bonds, which can probably be attributed to the significant steric hindrance generated by the large ${}^{t}$Bu and Ad groups. It is worth noting (Table 2) that despite the presence of large substituents, I${}^{t}$Bu${}_{2}$ and IAd${}_{2}$ form linear C⋯X halogen bonds without any exceptions.

**Figure 3:**
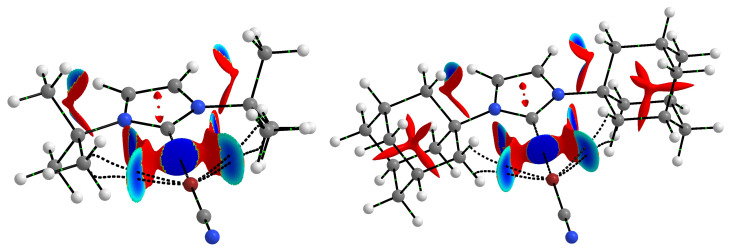
NCI-based *s*-isosurfaces (*s* = 0.5 a.u.) for the I${}^{t}$Bu${}_{2}\cdots$BrCN and IAd${}_{2}\cdots$BrCN complexes. Colors are coded according to a common $sgn(\lambda_{2})\rho$ scale (in a.u.): −0.0100, blue, −0.0075, cyan; −0.0050, green; −0.0025, yellow; and 0.0000, red. Cutoff of 0.050 a.u. was used for the electron density.

However, the steric hindrance of the bulky ${}^{t}$Bu and Ad groups does not seem to be the sole or even decisive reason for the long and relatively weak halogen bonds in the I${}^{t}$Bu${}_{2}$ and IAd${}_{2}$ complexes when we look at the NCI-based [124,125] *s*-isosurfaces of these complexes. Two representative examples (I${}^{t}$Bu${}_{2}\cdots$BrCN and IAd${}_{2}\cdots$BrCN) are shown in Figure 3.

In addition to the clearly visible areas of repulsion (in red) between the bromine atom and either the ${}^{t}$Bu or Ad groups, the subfigures obtained are characterized by the presence of as many as four bond paths between Br and either carbon (in the case of I${}^{t}$Bu${}_{2}\cdots$BrCN) or hydrogen (in the case of IAd${}_{2}\cdots$BrCN) atoms, which can be attributed to four C-H⋯Br hydrogen bonds. However, these bonds should be weaker than the C⋯Br halogen bond. The value of the electron density at the critical points of the C-H⋯Br hydrogen bonds is 0.009 a.u. (after using the formula of Emamian et al. [126] this would give the interaction energy of ca. −1.3 kcal/mol), while for the C⋯Br halogen bond, the value of $\rho_{C\cdots \mathrm{Br}}$ is 0.014 a.u. It is possible that the reason for the rather large C⋯Br distance in these two and similar complexes is the energetically favorable position of the bromine atom in the plane of both C-H bonds forming the C-H⋯Br hydrogen bonds. In other words, the bromine atom is on the prolongation of the C-H bond projections (see Figure 2). Thus, as Figure 3 shows, despite the steric effect of the ${}^{t}$Bu and Ad groups, these groups can also contribute to stabilization, and the resulting complex structure is due to a balance of different effects rather than just one of them. Furthermore, we also note the striking similarity of the NCI-based *s*-isosurfaces to complexes involving I${}^{t}$Bu${}_{2}$ and IAd${}_{2}$. The peculiarity is that the hydrogen bonds C-H⋯Br (more generally C-H⋯X) in question are characterized by significant values of bond ellipticity. In complexes I${}^{t}$Bu${}_{2}\cdots$BrCN and IAd${}_{2}\cdots$BrCN shown in Figure 3, these values are 15.5 and 7.2, respectively, but the value is as high as 148.7 in complex I${}^{t}$Bu${}_{2}\cdots$ClCN. Such large values of the bond ellipticity of the C-H⋯X hydrogen bonds result from the small distance between the BCPs of these bonds and the ring critical point (RCP) between them.

**Figure 4:**
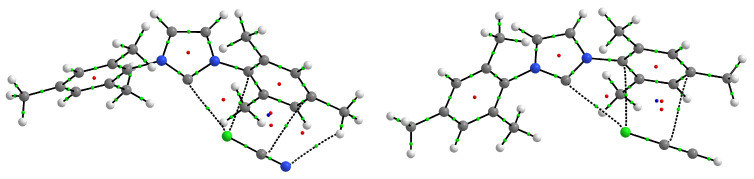
Molecular graphs of the IMes${}_{2}\cdots$ClCN and IMes${}_{2}\cdots$ClCCH complexes.

Interestingly, also the IMes${}_{2}\cdots$ClCN and IMes${}_{2}\cdots$ClCCH complexes are characterized by high $d_{C\cdots \mathrm{Cl}}$ values (3.168 and 3.359 Å, respectively), but in both cases the $D_{0}$ values are much higher (9.26 and 5.97 kcal/mol, respectively—which are also the largest $D_{0}$ values obtained for ClCN and ClCCH; see Table 2) than for the corresponding complexes with I${}^{t}$Bu${}_{2}$ or IAd${}_{2}$. Moreover, the $\rho_{C\cdots X}$ values are surprisingly small, only 0.011 and 0.008 a.u., respectively. A significant deviation from linearity (152.9${}^{\circ }$ and 154.6${}^{\circ }$, respectively) strongly suggests that the reason for such high dissociation energies in both of these complexes is the coexistence of other intermolecular interactions. Indeed, the molecular graphs (see Figure 4) show that ClCN and ClCCH in both complexes are located almost parallel to the ring of one of the mesityl groups and confirm the presence of various intermolecular interactions between these molecules and the Mes group.

**Figure 5:**
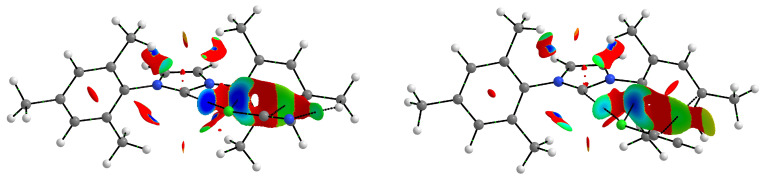
NCI-based *s*-isosurfaces (*s* = 0.5 a.u.) for the IMes${}_{2}\cdots$ClCN and IMes${}_{2}\cdots$ClCCH complexes. Colors are coded according to a common $sgn(\lambda_{2})\rho$ scale (in a.u.): −0.0100, blue; −0.0075, cyan; −0.0050, green; −0.0025, yellow; and 0.0000, red. Cutoff of 0.050 a.u. was used for the electron density.

In both cases, the Cl⋯C and C⋯C bond paths are present, which can be understood as the presence of an interaction between the Cl-C bond and the π-electron system of the mesityl group. In the case of the complex with ClCN, the C-H⋯N hydrogen bond path is additionally present. A more complete picture of the interaction between either ClCN or ClCCH and the mesityl group is provided by the NCI-based *s*-isosurfaces (Figure 5).

Indeed, they clearly show a large area of intermolecular interaction in this region. This region seamlessly merges with the region (on the left) of the interaction between the carbene carbon atom and the chlorine atom. In the case of the IMes${}_{2}\cdots$ClCN complex, it is clear that the Cl⋯C secondary interaction is similar in strength to the C⋯Cl halogen bond (the electron density values at the BCPs are 0.010 and 0.011 a.u., respectively) and that both should be much stronger than the C⋯C and C-H⋯N interactions ($\rho_{\mathrm{bcp}}$ values are 0.005 and 0.006 a.u., respectively). Exactly the same pattern of stabilizing interactions also occurs in the IMes${}_{2}\cdots$ClCCH complex; nevertheless, both C⋯Cl interactions should be weaker ($\rho_{\mathrm{bcp}}$ amounts to 0.008 and 0.009 a.u., respectively), though stronger than the C⋯C contacts (0.005 a.u.; one of them (rightmost) is not tracked by a bond path). Interestingly, therefore, it can be seen that in the case of the IMes${}_{2}\cdots$ClCCH complex, the C⋯Cl halogen bond should be weaker than the interaction between Cl and the mesityl carbon atom directly bonded to the nitrogen atom of the imidazol-2-ylidene ring.

**Figure 6:**
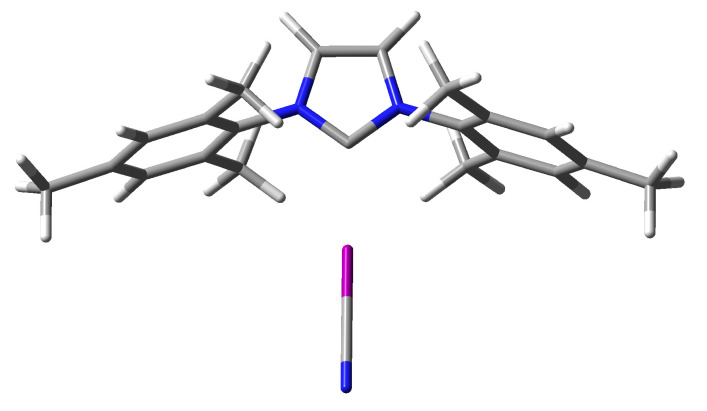
Structure of the IMes${}_{2}\cdots$ICN complex.

The $D_{0}$ values shown in Table 2 clearly indicate that of all the carbenes considered, IMes${}_{2}$ forms the strongest halogen bonds. Of course, taking into account the fact that the strongest halogen bond is formed by the iodine atom and the XCN molecule, it is not surprising that the strongest halogen bond occurs in the IMes${}_{2}\cdots$ICN complex (18.71 kcal/mol). Interestingly, despite the large size of the iodine atom, the strongest halogen bond in this complex corresponds to an extremely short C⋯I distance (only 2.541 Å), which is also the shortest halogen bond length in the complexes considered here. This bond is almost linear ($\alpha_{\mathrm{CXC}}$ = 179.4${}^{\circ }$). This complex is also characterized by the highest $\rho_{C\cdots X}$ value, amounting to as much as 0.053 a.u. The structure of the IMes${}_{2}\cdots$ICN complex is shown in Figure 6.

Replacing the iodine atom with a bromine atom and then a chlorine atom causes an increasing deviation from the linearity of the halogen bond, 176.2${}^{\circ }$ for Br, and as already mentioned, only 152.9${}^{\circ }$ for Cl. This result clearly shows that the chlorine atom has the least tendency to form linear or nearly linear halogen bonds. Of course, this is related to the weakest σ-hole. Importantly, it is clearly visible (Table 2) that in many cases, the IDipp${}_{2}$ carbene forms comparable to IMes${}_{2}$ strong halogen bonds. For example, in the case of the IMes${}_{2}\cdots$ICN complex (just discussed) and its counterpart IDipp${}_{2}\cdots$ICN, the dissociation energies are 18.71 and 17.56 kcal/mol, respectively. In the case of the BrCN counterparts, they are 11.18 and 10.95 kcal/mol, respectively, and in the case of BrCCH, the values of $D_{0}$ are 7.34 and 7.39 kcal/mol, respectively. It can, therefore, be concluded that despite the slightly different characteristics of the steric hindrance in the bay of the carbene carbon atom, both carbenes, i.e., IMes${}_{2}$ and IDipp${}_{2}$, form (similarly) strong halogen bonds.

It is worth noting that I${}^{i}$Pr${}_{2}$ also forms short and often strong halogen bonds. Importantly, the relatively small size of the ${}^{i}$Pr group causes that in the I${}^{i}$Pr${}_{2}\cdots$HD complexes the co-existence of other interactions is negligible and therefore the obtained dissociation energy can be identified with the energy of the resulting halogen bond. Of course, the shortest halogen bond is formed with ICN (only 2.552 Å), which is only 0.01 Å longer than in IMes${}_{2}\cdots$ICN. The $D_{0}$ value is 17.12 kcal/mol (Table 2).

The $\nabla^{2}\rho_{C\cdots X}$ values also listed in Table 2 show that the considered halogen bonds in the IR${}_{2}\cdots$XCN and IR${}_{2}\cdots$XCCH complexes are of the closed-shell type. All halogen bonds involving a chlorine or bromine atom have a (small) positive $H_{C\cdots X}$ value. However, this characteristic changes fundamentally for most halogen bonds involving an iodine atom. Namely, this value becomes negative, showing that the given C⋯I halogen bond has a partial covalent character. The most negative $H_{C\cdots X}$ values are characteristic of the IMes${}_{2}\cdots$ICN and I${}^{i}$Pr${}_{2}\cdots$ICN complexes (−0.0109 and −0.0108 a.u., respectively), which also feature similar values of other parameters describing the halogen bond (Table 2). Quite the opposite, for the complexes involving ICN, I${}^{t}$Bu${}_{2}$ and IAd${}_{2}$ are the only carbenes giving (slightly) positive $H_{C\cdots X}$ values (0.0006 a.u.). This is an indirect confirmation of the relative weakness of the I${}^{t}$Bu${}_{2}\cdots$ICN and IAd${}_{2}\cdots$ICN complexes.

### 2.3. Relationships between Physical Quantities

**Figure 7:**
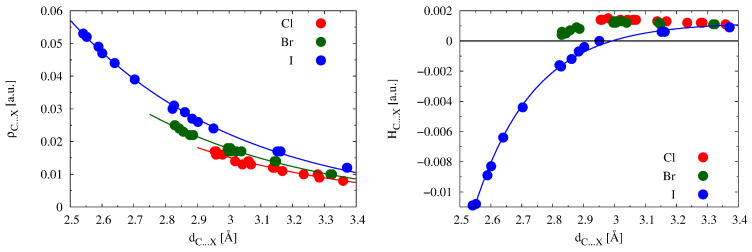
Relationship between either $\rho_{C\cdots X}$ (**left**) or $H_{C\cdots X}$ (**right**) and $d_{C\cdots X}$ obtained for the IR${}_{2}\cdots$XCN and IR${}_{2}\cdots$XCCH (X = Cl, Br, I) complexes.

Relatively often in the literature on non-covalent interactions, one can find a statement that the electron density at BCP of a non-covalent bond is linearly correlated with the length of this bond. For this reason, it was tempting to check whether this type of relationship occurs in the C⋯X halogen bonds in the IR${}_{2}\cdots$XCN and IR${}_{2}\cdots$XCCH complexes studied here. Of course, this type of correlation was not expected to be perfect since the presence of various accompanying interactions in many of the complexes to some extent affects the value of the C⋯X distance. Indeed, the coefficient of determination $R^{2}$ is only 0.832 when all systems are considered together. However, the left subfigure in Figure 7 clearly shows that the reason for such a small value of $R^{2}$ is that the corresponding points for the relationship $\rho_{C\cdots X}$ vs. $d_{C\cdots X}$ are distributed exponentially rather than linearly. Moreover, systems that differ in the type of X should be considered separately.

Of course, the corresponding curve for Br is above the curve for Cl, and above it is the curve for I. This is due to the general strength of the halogen bonds Cl < Br < I, as mentioned at the beginning of Section 2.2. It is also clear from Figure 7 that the C⋯Cl halogen bonds are relatively long and with a fairly short range of values, while the length range is larger for C⋯Br, but both of these ranges are definitely overcome by C⋯I, the range of which is up to 0.83 Å, from 2.541 Å to 3.372 Å. However, most importantly in the context of the left subfigure in Figure 7, it is only for small ranges of $d_{C\cdots X}$ (or $\rho_{C\cdots X}$) values that it makes sense to look for reasonably good linear correlations.

In turn, the right subfigure in Figure 7 shows the exponential relationship between $H_{C\cdots X}$ and $d_{C\cdots X}$ determined for complexes where X = I. It is clear that similar relationships should be determined for different X separately. This plot is a nice illustration of the previously discussed $H_{C\cdots X}$ values presented in Table 2; namely, all the complexes with Cl or Br feature positive values of $H_{C\cdots X}$. If X = I, then most complexes are in the range ±0.002 a.u. However, the characteristics of the relationship between $H_{C\cdots X}$ and $d_{C\cdots X}$ cause that with the decrease in the distance C⋯I, the value of $H_{C\cdots X}$ decreases rapidly, becoming a significantly negative number, which indicates a significant covalent contribution of the interaction. In extreme cases, an [IR${}_{2}$X]${}^{+}$[D]${}^{-}$ ionic pair may be formed [95].

We now show that the $R^{2}$ values can also be used to find the carbenes that ’break’ the overall linear correlation the most, which is most likely explained by the presence of various secondary interactions whose overall contribution to the complex dissociation energy may be non-negligible. For this purpose, the relationship between $d_{C\cdots X}$ and $D_{0}$ was investigated. Of course, as expected, the value of $R^{2}$ is small (0.652), indicating that the relationship between $d_{C\cdots X}$ and $D_{0}$ is far from linear. However, it is interesting to analyze the obtained $R^{2}$ values when linear correlations are made for each of the carbenes separately (see Table 3).

As is clearly seen, the value of $R^{2}$ is by far the smallest for I${}^{t}$Bu${}_{2}$ (0.255) and IAd${}_{2}$ (0.274). Indeed, both of these carbenes are characterized by the presence of bulky substituents, which, in addition to steric hindrance, can form four hydrogen bonds of the C-H⋯X type, as exemplified by the I${}^{t}$Bu${}_{2}\cdots$BrCN and IAd${}_{2}\cdots$BrCN complexes shown in Figure 3. Next in line is IMes${}_{2}$ (0.828), and indeed, this carbene is also prone to creating additional accompanying interactions—the best examples of which are the IMes${}_{2}\cdots$ClCN and IMes${}_{2}\cdots$ClCCH complexes shown in Figure 4 and Figure 5. A similar analysis was also performed on the X-donor molecule: ClCN (0.099), BrCN (0.713), ICN (0.904), ClCCH (0.417), BrCCH (0.522), ICCH (0.842). The obtained $R^{2}$ values suggest that the ClCN molecule is the most ’problematic’ and that the quality of the linear correlation between $d_{C\cdots X}$ and $D_{0}$ improves in the order Cl → Br → I, which of course reflects the increasingly developed σ-hole on the halogen atom.

### 2.4. Halogen Bond vs. Hydrogen Bond

It is instructive to compare the halogen bond characterization discussed here with the hydrogen bond characterization obtained earlier [65] for the same set of carbenes. As has been shown [65], IDipp${}_{2}$ is prone to forming the strongest complexes with the proton-donor molecule, which was attributed to the co-presence (apart from the dominant (carbene)C⋯H hydrogen bond) of multiple accompanying interactions, such as numerous, e.g., C-H⋯Y hydrogen bonds or C-H⋯H-C contacts, depending on the type of proton-donor molecule. Although the individual interactions are rather very weak, a large number of them can significantly affect the overall strength of the complex. The strongest complex was IDipp${}_{2}\cdots$HF with the dissociation energy of 19.9 kcal/mol (ωB97X-D/6-311++G(d,p)), which is only slightly higher than the strongest halogen bond obtained for the IMes${}_{2}\cdots$ICN complex (ca. 18.7 kcal/mol).

Importantly, it has been shown previously [65] that replacing both hydrogen atoms in the N-H bonds of imidazol-2-ylidene with any substituent under consideration led to a stronger complex with HF, HCN, H${}_{2}$O, or MeOH. Although this result is in agreement with the lowest nucleophilicity index obtained for I (Table 1), as already discussed, for IR${}_{2}\cdots$XD complexes, weaker halogen bonds are generally formed by the I${}^{t}$Bu${}_{2}$ carbene (Table 2). The only exception are systems with chlorine, for which the dissociation energy for I is actually (slightly) lower. For example, for the complexes I⋯ClCN and I${}^{t}$Bu${}_{2}\cdots$ClCN, the $D_{0}$ values are 5.87 and 6.11 kcal/mol, respectively. Apparently, the presence of branched tert-butyl groups hinders the access of larger halogens to the carbene lone electron pair, changing this relationship to the opposite. For example, in the case of ICN, the dissociation energy for I amounts to 14.3 kcal/mol, whereas for I${}^{t}$Bu${}_{2}$, this energy is only 9.7 kcal/mol.

### 2.5. Crystal Structures

**Figure 8:**
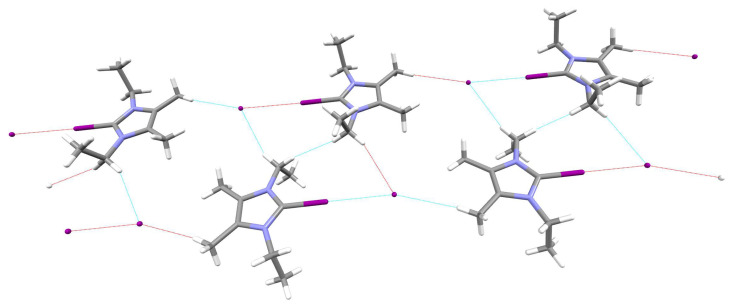
Fragment of the crystal lattice of HARXAZ, i.e., 1,3-diethyl-2-iodo-4,5-dimethylimidazolium iodide. The carbene carbon atom forms a C–I⋯I${}^{-}$ link.

In many cases, the interaction of the carbene carbon atom with the halogen atom (especially iodine) is so strong that a C–X bond is formed. An example is 1,3-diethyl-2-iodo-4,5-dimethylimidazolium iodide (HARXAZ) [88], whose lattice fragment is shown in Figure 8. In this case, the carbene carbon forms a C–I⋯I${}^{-}$ link.

**Figure 9:**
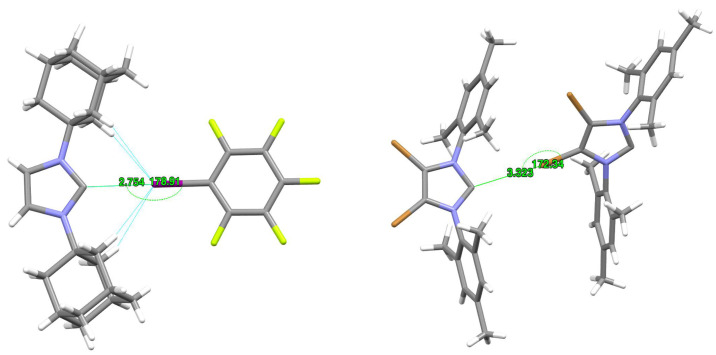
**Left**: fragment of YABJAM, i.e., (2-(1,3-diadamantylimidazolium))pentafluorophenyl-$\lambda^{3}$-iodanide tetrahydrofuran solvate (tetrahydrofuran solvate has been removed for better clarity of the figure); **right**: lattice fragment of 4,5-dibromo-1,3-bis(2,4,6-trimethylphenyl)imidazol-2-ylidene (AHEVEO).

However, using CSD [127], we also managed to find systems in which the presence of a halogen bond to the carbene carbon atom is undeniable. Two such examples are shown in Figure 9.

On the left side of Figure 9, a lattice fragment of the previously mentioned (2-(1,3-diadamantylimidazolium))pentafluorophenyl-$\lambda^{3}$-iodanide tetrahydrofuran solvate present in CSD as YABJAM [87] is shown. In this system, IAd${}_{2}$ forms a C⋯I type halogen bond with pentafluoroiodophenyl. The contact length of C⋯I is only 2.754 Å, much shorter than the theoretical length of halogen bonds in IAd${}_{2}\cdots$ICN and IAd${}_{2}\cdots$ICCH (3.152 and 3.371 Å, respectively; Table 2). The C⋯I–C bridge in this system is almost linear (178.91${}^{\circ }$). On the right side of Figure 9, a fragment of the crystal lattice of 4,5-dibromo-1,3-bis(2,4,6-trimethylphenyl)imidazol-2-ylidene (AHEVEO) [128] is shown. In this case, the individual molecules are connected by a C⋯Br halogen bond. It is much longer (3.323 Å) than the theoretically determined C⋯Br halogen bond lengths in the IMes${}_{2}\cdots$BrCN (2.830 Å) and IMes${}_{2}\cdots$BrCCH (2.999 Å) complexes (Table 2). The C⋯Br-C bridge in AHEVEO forms an angle of 172.34${}^{\circ }$.

## 3. Materials and Methods

Geometries of all the systems (for which charge = 0 and multiplicity = 1) were fully optimized using the global hybrid meta-GGA M06-2X exchange-correlation functional [129,130] of Density Functional Theory [131,132,133]. Importantly, the M06-2X functional was shown to give accurate geometries and dissociation energies of halogen bonds [134]. Moreover, as recently shown [135], this functional also gives reasonable values for the electron density at the bond critical point—the most important topological parameter in the QTAIM-based [123] analysis. The 6-311++G(d,p) basis set being of the triple-zeta type and containing both polarization and diffuse functions on all atoms [136,137,138,139,140] was used for all atoms but iodine for which the Karlsruhe def2-TZVPD basis set [141,142,143,144,145], taken from Basis Set Exchange (BSE) [146], was used instead. The absence of imaginary frequencies indicated true equilibrium structures every time. Both the geometry optimization and the frequency analysis were performed using the Gaussian 16 (Revision C.01) program [147].

The nucleophilicity index was calculated by four methods (Method I–IV) [122]:

(1) $$\mathrm{Method}\;I:\;\;\;\;\;N_{I}=E_{\mathrm{HOMO}}-E_{\mathrm{HOMO}}^{\mathrm{TCE}}\;$$

(2) $$\mathrm{Method}\;\mathrm{II}:\;\;\;\;\;N_{\mathrm{II}}=\frac{1}{\omega }\;\;\mathrm{where}\;\;\;\omega =\frac{\mu^{2}}{2\eta }\;$$

(3) $$\;\;\;\;\;\mathrm{Method}\;\mathrm{III}:\;\;\;\;\;N_{\mathrm{III}}=\frac{10}{\omega^{-}}\;\;\mathrm{where}\;\;\;\omega^{-}=\frac{I^{2}}{2(I-A)}\;$$

(4) $$\;\;\;\;\;\mathrm{Method}\;\mathrm{IV}:\;\;\;\;\;N_{\mathrm{IV}}=\frac{10}{\omega^{-}}\;\;\mathrm{where}\;\;\;\omega^{-}=\frac{{\left(3I+A\right)}^{2}}{16(I-A)}$$

According to Method I [148], the nucleophilicity index ($N_{I}$) is simply the difference between the HOMO energies of the test molecule and the reference tetracyanoethylene (TCE) molecule [149,150]. According to Method II, the nucleophilicity index ($N_{\mathrm{II}}$) is determined as the inverse [151] of the electrophilicity index ω [152], where μ is the electronic chemical potential and η is the chemical hardness. Both of these physical quantities were calculated within the finite difference approximation, where $\mu =-\frac{1}{2}\left(I+A\right)$ and η=I−A. Somewhat similarly, Methods III and IV use the inverse of the so-called electrodonating power $\omega^{-}$ [153]. The values of the ionization potential (*I*) and the electron affinity (*A*), which were needed to determine the nucleophilicity index according to Methods II–IV, were calculated in the vertical approximation [154], i.e., using the total energies of cations and anions having geometries of neutral carbenes. This approach is much more reasonable than using HOMO and LUMO energies for this purpose. It should be noted that according to Methods II–IV, the unit of the nucleophilicity index is eV${}^{-1}$ and eV when the simplest Method I is used.

The dissociation energy was calculated as the difference between the total energy of a complex and the sum of the total energies of isolated subsystems in their own fully optimized structures. Then, the total energies were corrected for the zero-point vibrational energies (ZPVE). Dissociation energies are given as positive values.

Topological analysis of the considered complexes was performed on the basis of Bader’s Quantum Theory of Atoms in Molecules (QTAIM) [123]. In addition, this analysis was supplemented with a non-local analysis of weak non-covalent interactions according to the Noncovalent Interaction (NCI) method [124,125]. This method is based on the reduced electron density gradient ($s=1/\left(2{\left(3\pi^{2}\right)}^{1/3}\right)\left|\nabla \rho \right|/\rho^{4/3}$) and $sgn(\lambda_{2})\rho$, i.e., the electron density multiplied by the sign of the second eigenvalue of the electron density Hessian matrix ($\lambda_{2}$). As a consequence, NCI allows for displaying individual weak interactions as certain regions of real space rather than as local features of a BCP corresponding to a pairwise interatomic contact. Then, most importantly, these interactions can be easily and visually (by using different colors) separated into attractive (if $\lambda_{2}<0$) and repulsive (if $\lambda_{2}>0$). A common color scale (in a.u.) was used for all systems when producing the NCI-based *s*-isosurfaces: −0.0100, blue; −0.0075, cyan; −0.0050, green; −0.0025, yellow; and 0.0000, red. The QTAIM- and NCI-based calculations were performed using the AIMAll program [155].

## 4. Conclusions

The article describes halogen bonds involving the carbene carbon atom of imidazol-2-ylidene (I) or its derivatives (IR${}_{2}$) as a lone electron pair donor. Gradually larger substituents of great experimental importance were taken as R: methyl = Me, *iso*-propyl = ${}^{i}$Pr, *tert*-butyl = ${}^{t}$Bu, phenyl = Ph, mesityl = Mes, 2,6-diisopropylphenyl = Dipp, 1-adamantyl = Ad. In turn, the halogen atom donors were XCN and XCCH molecules, where X = Cl, Br, I. As a consequence of such a set of carbenes and X-donor molecules, the structures of 48 dimes of the IR${}_{2}\cdots$XCN and IR${}_{2}\cdots$XCCH type, bound by the thematic halogen bond C${}_{\mathrm{carbene}}\cdots$X, have been investigated.

It has been shown that, for a given X-donor, the halogen bond strength increases in the order Cl < Br < I and that the XCN molecule forms stronger complexes than XCCH. It has also been shown that of all the carbenes considered, IMes${}_{2}$ forms the strongest and also the shortest halogen bonds. Of course, taking into account the above, the strongest ($D_{0}$ = 18.71 kcal/mol) and at the same time the shortest ($d_{C\cdots I}$ = 2.541 Å despite the presence of a large iodide atom) halogen bond has been found in the IMes${}_{2}\cdots$ICN complex. In many cases, IDipp${}_{2}$ forms as strong halogen bonds as IMes${}_{2}$.

In some cases, an extreme example being the complexes IMes${}_{2}\cdots$ClCN and IMes${}_{2}\cdots$ClCCH, various co-existing secondary interactions are possible, which result in significant non-linearity of the halogen bond. As expected, this tendency is greatest when X = Cl that can be related to the weakest σ-hole. Noteworthy is the fact that I${}^{i}$Pr${}_{2}$ forms short and often strong halogen bonds, practically without the participation of additional secondary interactions. For example, in the $I^{i}$Pr${}_{2}\cdots$ICN complex, $D_{0}$ = 17.12 kcal/mol and $d_{C\cdots I}$ = 2.552 Å only.

Based on the computed values of the nucleophilicity index, I${}^{t}$Bu${}_{2}$ and IDipp${}_{2}$ should form the strongest halogen bonds. Surprisingly, however, it has turned out that I${}^{t}$Bu${}_{2}$ gives the weakest and not the strongest complexes if X ≠ Cl. This carbene also forms the longest and always linear halogen bonds. While this finding could easily be attributed to the significant steric hindrance produced by the branched *tert*-butyl groups, it has been shown that an equally important reason could be the possibility of additional stabilization of the complex through the formation of four weak C-H⋯X hydrogen bonds. The same is true for complexes with IAd${}_{2}$, which also forms long halogen bonds.

It has also been shown that the relationship between the value of the electron density at the critical point of the C⋯X halogen bond and its length is not linear but rather exponential and only for small ranges of either of these parameters it makes sense to look for a linear relationship. In addition, the relationship should be determined separately for different types of atom X.

## Figures and Tables

**Table 1:** Nucleophilicity index of the analyzed carbenes.

	Molecule							
Method	I	IMe${}_{2}$	I${}^{i}$Pr${}_{2}$	I${}^{t}$Bu${}_{2}$	IPh${}_{2}$	IMes${}_{2}$	IDipp${}_{2}$	IAd${}_{2}$
I	3.27	3.39	3.42	3.63	3.28	3.37	3.84	3.24
II	1.22	1.30	1.31	1.36	1.28	1.30	1.25	1.32
III	2.54	2.67	2.74	2.84	2.67	2.75	2.89	2.72
IV	2.39	2.53	2.58	2.68	2.52	2.58	2.68	2.53

**Table 2:** Dissociation energy ($D_{0}$ in kcal/mol), halogen bond length ($d_{C\cdots X}$ in Å), C-X-C angle ($\alpha_{\mathrm{CXC}}$ in degrees), electron density ($\rho_{C\cdots X}$ in a.u.), Laplacian of the electron density ($\nabla^{2}\rho_{C\cdots X}$ in a.u.), and the total electronic energy density ($H_{C\cdots X}$ in a.u.) obtained for the IR${}_{2}\cdots$XCCH and IR${}_{2}\cdots$XCN (X = Cl, Br, I) complexes.

X-Donor	Parameters	Carbene							
		I	IMe${}_{2}$	I${}^{i}$Pr${}_{2}$	I${}^{t}$Bu${}_{2}$	IPh${}_{2}$	IMes${}_{2}$	IDipp${}_{2}$	IAd${}_{2}$
ClCN	$D_{0}$	5.87	6.63	6.99	6.11	6.99	9.26	8.59	6.78
	$d_{C\cdots X}$	2.979	2.959	2.955	3.144	2.959	3.168	3.019	3.138
	$\alpha_{\mathrm{CXC}}$	180.0	180.0	180.0	180.0	179.4	152.9	171.7	180.0
	$\rho_{C\cdots X}$	0.016	0.017	0.017	0.012	0.016	0.011	0.014	0.012
	$\nabla^{2}\rho_{C\cdots X}$	0.050	0.052	0.052	0.037	0.052	0.035	0.045	0.038
	$H_{C\cdots X}$	0.0015	0.0014	0.0014	0.0012	0.0014	0.0013	0.0014	0.0013
BrCN	$D_{0}$	8.58	9.59	10.00	7.23	9.37	11.18	10.95	7.84
	$d_{C\cdots X}$	2.877	2.843	2.829	3.148	2.887	2.830	2.856	3.143
	$\alpha_{\mathrm{CXC}}$	180.0	180.0	180.0	180.0	179.2	176.2	177.0	180.0
	$\rho_{C\cdots X}$	0.022	0.024	0.025	0.014	0.022	0.025	0.023	0.014
	$\nabla^{2}\rho_{C\cdots X}$	0.061	0.063	0.064	0.039	0.060	0.065	0.062	0.039
	$H_{C\cdots X}$	0.0009	0.0005	0.0004	0.0011	0.0008	0.0006	0.0007	0.0012
ICN	$D_{0}$	14.31	16.18	17.12	9.71	14.81	18.71	17.56	10.54
	$d_{C\cdots X}$	2.640	2.589	2.552	3.162	2.703	2.541	2.601	3.152
	$\alpha_{\mathrm{CXC}}$	180.0	180.0	178.2	180.0	177.8	179.4	180.0	180.0
	$\rho_{C\cdots X}$	0.044	0.049	0.052	0.017	0.039	0.053	0.047	0.017
	$\nabla^{2}\rho_{C\cdots X}$	0.074	0.073	0.073	0.041	0.071	0.075	0.073	0.041
	$H_{C\cdots X}$	−0.0064	−0.0089	−0.0108	0.0006	−0.0044	−0.0109	−0.0083	0.0006
ClCCH	$D_{0}$	3.43	4.02	4.29	3.93	4.33	5.97	5.90	4.47
	$d_{C\cdots X}$	3.042	3.062	3.060	3.279	3.069	3.359	3.235	3.284
	$\alpha_{\mathrm{CXC}}$	165.9	180.0	180.0	180.0	179.3	154.6	164.3	180.0
	$\rho_{C\cdots X}$	0.013	0.014	0.014	0.010	0.013	0.008	0.010	0.009
	$\nabla^{2}\rho_{C\cdots X}$	0.043	0.044	0.044	0.030	0.043	0.024	0.030	0.029
	$H_{C\cdots X}$	0.0014	0.0014	0.0014	0.0012	0.0014	0.0011	0.0012	0.0012
BrCCH	$D_{0}$	5.30	6.16	6.40	4.70	6.09	7.34	7.39	5.32
	$d_{C\cdots X}$	3.023	3.004	2.996	3.319	3.039	2.999	3.006	3.324
	$\alpha_{\mathrm{CXC}}$	177.4	180.0	180.0	180.0	179.9	174.8	177.2	180.0
	$\rho_{C\cdots X}$	0.017	0.018	0.018	0.010	0.017	0.017	0.017	0.010
	$\nabla^{2}\rho_{C\cdots X}$	0.049	0.051	0.051	0.029	0.048	0.051	0.051	0.029
	$H_{C\cdots X}$	0.0013	0.0012	0.0012	0.0011	0.0012	0.0013	0.0013	0.0011
ICCH	$D_{0}$	9.15	10.47	11.01	6.62	9.77	12.05	11.66	7.35
	$d_{C\cdots X}$	2.902	2.861	2.827	3.372	2.951	2.822	2.884	3.371
	$\alpha_{\mathrm{CXC}}$	178.7	180.0	177.7	180.0	178.3	177.9	179.5	180.0
	$\rho_{C\cdots X}$	0.026	0.029	0.031	0.012	0.024	0.030	0.027	0.012
	$\nabla^{2}\rho_{C\cdots X}$	0.063	0.065	0.066	0.030	0.059	0.068	0.064	0.030
	$H_{C\cdots X}$	−0.0004	−0.0012	−0.0017	0.0009	0.0000	−0.0016	−0.0007	0.0009

**Table 3:** Coefficient of determination ($R^{2}$) for the relationship between $d_{C\cdots X}$ and $D_{0}$ depending on the carbene.

Carbene							
I	IMe${}_{2}$	I${}^{i}$Pr${}_{2}$	I${}^{t}$Bu${}_{2}$	IPh${}_{2}$	IMes${}_{2}$	IDipp${}_{2}$	IAd${}_{2}$
0.964	0.984	0.993	0.255	0.937	0.828	0.932	0.274

## Data Availability

The fully optimized structures of the investigated complexes are included in Supplementary Materials. Other data are available from the author upon reasonable request.

## Supplementary Materials

The following are available at https://www.mdpi.com/article/10.3390/ijms24109057/s1: the fully optimized structures of the investigated complexes.

## Abbreviations

The following abbreviations are used in this manuscript:

XB	halogen bond
XD	halogen-donor
NHC	N-heterocyclic-carbenes
I	imidazol-2-ylidene
IR${}_{2}$	R-substituted imidazol-2-ylidene
Me	methyl group
${}^{i}$Pr	isopropyl group
${}^{t}$Bu	*tert*-butyl group
Ph	phenyl group
Mes	mesityl group
Dipp	2,6-diisopropylphenyl group
Ad	adamantyl group
DFT	Density Functional Theory
QTAIM	Quantum Theory of Atoms in Molecules
BCP	bond critical point
RCP	ring critical point
NCI	Noncovalent Interaction (index)
