# Supplementary material for: Halogen Bond to Experimentally Significant N-Heterocyclic Carbenes (I, IMe2, IiPr2, ItBu2, IPh2, IMes2, IDipp2, IAd2; I = Imidazol-2-ylidene)
Source: Int J Mol Sci. 2023 May 21;24(10):9057. doi: 10.3390/ijms24109057 (PMC10219477; doi:10.3390/ijms24109057)
Supplement: Supplementary file 1 [file ijms-24-09057-s001.zip › ijms-2390129-supplementary.pdf]

Halogen Bond to Experimentally Significant N-Heterocyclic Carbenes (I, IMe2, Ii Pr2, It Bu2, IPh2, IMes2, IDipp2, IAd2; I = imidazol-2-ylidene)

Mirosław Jabłoński

Faculty of Chemistry, Nicolaus Copernicus University in Torun, Gagarina 7, 87-100 Torun, Poland;
teojab@chem.umk.pl; Tel.: +48-056-611-4695

SUPPORTING INFORMATION

I-ClCN

7	-0.000000	1.048352	-1.974041
6	0.000000	-0.000000	-1.109247
7	-0.000000	-1.048352	-1.974041
6	0.000000	-0.675122	-3.309507
6	0.000000	0.675122	-3.309507
1	-0.000000	1.379042	-4.123234
1	-0.000000	-1.379042	-4.123234
1	-0.000000	2.002009	-1.651508
1	-0.000000	-2.002009	-1.651508
6	0.000000	0.000000	3.516444
17	0.000000	0.000000	1.869788
7	0.000000	0.000000	4.667221

I-BrCN

7	-0.000000	1.049418	-2.340484
6	-0.000000	-0.000000	-1.480481
7	-0.000000	-1.049418	-2.340484
6	-0.000000	-0.675206	-3.675266
6	0.000000	0.675206	-3.675266
1	-0.000000	1.379458	-4.488632
1	-0.000000	-1.379458	-4.488632
1	-0.000000	2.002960	-2.016495
1	-0.000000	-2.002960	-2.016495
6	0.000000	0.000000	3.216303
35	0.000000	0.000000	1.396861
7	0.000000	0.000000	4.367879

I-ICN

7	0.000000	1.052577	-2.459037
6	0.000000	-0.000000	-1.612315
7	-0.000000	-1.052577	-2.459037
6	0.000000	-0.675446	-3.792286
6	0.000000	0.675446	-3.792286
1	0.000000	1.380033	-4.605177
1	-0.000000	-1.380033	-4.605177
1	0.000000	2.005517	-2.130054
1	-0.000000	-2.005517	-2.130054
6	0.000000	0.000000	3.118025
53	0.000000	0.000000	1.027730
7	0.000000	0.000000	4.271495

I-ClCCH

7	1.289716	0.946202	0.000000
6	0.000000	1.378414	0.000000
7	0.182607	2.725843	0.000000
6	1.514020	3.114742	0.000000
6	2.227139	1.967895	0.000000
1	3.290010	1.800284	0.000000
1	1.833674	4.142215	0.000000
1	1.515908	-0.035723	0.000000
1	-0.595680	3.363921	0.000000
6	-1.381557	-3.071140	0.000000
6	-1.546981	-4.258458	0.000000
1	-1.698088	-5.310702	0.000000
17	-1.148694	-1.438413	0.000000

I-BrCCH

7	1.173341	-2.508691	-0.000000
6	0.188071	-1.573263	-0.000000
7	-0.918776	-2.361514	-0.000000
6	-0.640150	-3.719726	-0.000000
6	0.706935	-3.814573	-0.000000
1	1.351816	-4.675845	-0.000000
1	-1.399272	-4.482232	-0.000000
1	2.146700	-2.251762	-0.000000
1	-1.846516	-1.970024	-0.000000
6	-0.195678	3.242860	0.000000
6	-0.325155	4.436842	0.000000
1	-0.438825	5.494159	0.000000
35	0.000000	1.444123	0.000000

I-ICCH

7	-0.980092	-2.606860	-0.000000
6	0.096649	-1.783143	0.000000
7	1.118868	-2.673625	0.000000
6	0.701783	-3.995570	0.000000
6	-0.648192	-3.952600	-0.000000
1	-1.377788	-4.743297	-0.000000
1	1.379680	-4.831017	0.000000
1	-1.922055	-2.249609	-0.000000
1	2.081629	-2.377759	0.000000
6	-0.114671	3.143326	-0.000000
6	-0.183680	4.345932	-0.000000
1	-0.244232	5.408347	-0.000000
53	-0.000000	1.117152	0.000000

IMe2-ClCN

7	-0.000000	1.059672	-1.675951
6	0.000000	0.000000	-0.823777
7	-0.000000	-1.059672	-1.675951
6	0.000000	-0.676056	-3.005746
6	0.000000	0.676056	-3.005746
1	-0.000000	1.381466	-3.819986
1	-0.000000	-1.381466	-3.819986
6	0.000000	-0.000000	3.784306
6	-0.000000	2.442160	-1.231631
1	0.890766	2.958590	-1.592874
1	-0.890766	2.958590	-1.592874
1	-0.000000	2.439116	-0.144201
6	-0.000000	-2.442160	-1.231631
1	-0.890766	-2.958590	-1.592874
1	0.890766	-2.958590	-1.592874
1	-0.000000	-2.439116	-0.144201
17	0.000000	-0.000000	2.135441
7	0.000000	-0.000000	4.935148

IMe2-BrCN

7	0.000000	1.060929	-2.024776
6	-0.000000	0.000000	-1.177832
7	-0.000000	-1.060929	-2.024776
6	-0.000000	-0.676117	-3.353873
6	0.000000	0.676117	-3.353873
1	0.000000	1.381896	-4.167668
1	-0.000000	-1.381896	-4.167668
6	0.000000	-0.000000	3.490927
6	0.000000	2.444299	-1.579997
1	0.890935	2.959111	-1.942356
1	-0.890935	2.959111	-1.942356
1	0.000000	2.444276	-0.492560
6	-0.000000	-2.444299	-1.579997
1	-0.890935	-2.959111	-1.942356
1	0.890935	-2.959111	-1.942356
1	-0.000000	-2.444276	-0.492560
35	0.000000	-0.000000	1.664757
7	0.000000	-0.000000	4.642591

IMe2-ICN

7	0.000000	1.064955	-2.160280
6	0.000000	0.000000	-1.326581
7	-0.000000	-1.064955	-2.160280
6	-0.000000	-0.676179	-3.487321
6	0.000000	0.676179	-3.487321
1	0.000000	1.382917	-4.300031
1	-0.000000	-1.382917	-4.300031
6	-0.000000	-0.000000	3.376611
6	0.000000	2.452783	-1.719795
1	0.891514	2.961127	-2.087936

1	-0.891514	2.961127	-2.087936
1	0.000000	2.465040	-0.632555
6	-0.000000	-2.452783	-1.719795
1	-0.891514	-2.961127	-2.087936
1	0.891514	-2.961127	-2.087936
1	-0.000000	-2.465040	-0.632555
53	-0.000000	-0.000000	1.262876
7	-0.000000	-0.000000	4.530515

IMe2-ClCCH

7	-1.059077	1.732623	0.000000
6	0.000000	0.878339	0.000000
7	1.059306	1.732384	-0.000000
6	0.676421	3.062607	-0.000000
6	-0.675893	3.062776	0.000000
1	-1.380865	3.877489	0.000000
1	1.381562	3.877174	-0.000000
6	-0.000224	-3.833929	-0.000000
6	-0.000236	-5.033164	-0.000000
1	-0.000227	-6.096092	-0.000000
6	-2.439931	1.284725	0.000000
1	-2.958076	1.644109	0.890771
1	-2.958076	1.644109	-0.890771
1	-2.430875	0.197173	0.000000
6	2.440038	1.284095	-0.000000
1	2.958285	1.643333	-0.890770
1	2.958285	1.643333	0.890770
1	2.430657	0.196545	-0.000000
17	-0.000195	-2.183230	-0.000000

IMe2-BrCCH

7	-0.000000	1.059856	-2.117456
6	0.000000	-0.000000	-1.266152
7	-0.000000	-1.059856	-2.117456
6	0.000000	-0.676033	-3.447409
6	0.000000	0.676033	-3.447409
1	-0.000000	1.381588	-4.261514
1	0.000000	-1.381588	-4.261514
6	-0.000000	0.000000	3.550025
6	-0.000000	0.000000	4.751109
1	-0.000000	0.000000	5.814562
6	-0.000000	2.441242	-1.669290
1	0.890744	2.958585	-2.029342
1	-0.890745	2.958585	-2.029341
1	0.000000	2.434287	-0.581789
6	0.000000	-2.441242	-1.669290
1	-0.890744	-2.958585	-2.029342
1	0.890745	-2.958585	-2.029341
1	-0.000000	-2.434287	-0.581789
35	-0.000000	0.000000	1.737837

IMe2-ICCH

7	-0.000000	1.061666	-2.323298
6	0.000000	0.000000	-1.478641
7	-0.000000	-1.061666	-2.323298
6	0.000000	-0.676184	-3.651987
6	-0.000000	0.676184	-3.651987
1	-0.000000	1.382113	-4.465627
1	0.000000	-1.382113	-4.465627
6	0.000000	-0.000000	3.420650
6	0.000000	-0.000000	4.625594
1	0.000000	-0.000000	5.689800
6	-0.000000	2.445518	-1.878018
1	0.891132	2.959301	-2.241146
1	-0.891133	2.959300	-2.241146
1	0.000000	2.446502	-0.790357
6	0.000000	-2.445518	-1.878018
1	-0.891132	-2.959301	-2.241146
1	0.891133	-2.959300	-2.241146
1	-0.000000	-2.446502	-0.790357
53	0.000000	-0.000000	1.382403

IiPr2-ClCN

7	0.000000	1.061799	-1.191634
6	0.000000	0.000000	-0.342493
7	-0.000000	-1.061799	-1.191634
6	0.000000	-0.676473	-2.521293
6	0.000000	0.676473	-2.521293
1	0.000000	1.374080	-3.342091
1	-0.000000	-1.374080	-3.342091
6	-0.000000	0.000000	4.261810
17	-0.000000	0.000000	2.612354
6	0.000000	-2.452892	-0.731143
6	1.266753	-3.169767	-1.187697
6	-1.266753	-3.169767	-1.187697
1	0.000000	-2.374442	0.357233
1	2.154444	-2.630499	-0.853800
1	1.294452	-4.180253	-0.775542
1	1.299285	-3.250401	-2.277631
1	-2.154444	-2.630499	-0.853800
1	-1.299285	-3.250401	-2.277631
1	-1.294452	-4.180253	-0.775542
6	0.000000	2.452892	-0.731143
6	-1.266753	3.169767	-1.187697
6	1.266753	3.169767	-1.187697
1	0.000000	2.374442	0.357233
1	-2.154444	2.630499	-0.853800
1	-1.294452	4.180253	-0.775542
1	-1.299285	3.250401	-2.277631
1	2.154444	2.630499	-0.853800
1	1.299285	3.250401	-2.277631
1	1.294452	4.180253	-0.775542
7	-0.000000	0.000000	5.412646

IiPr2-BrCN

7	0.000000	1.063125	-1.517288
6	0.000000	0.000000	-0.673611
7	-0.000000	-1.063125	-1.517288
6	0.000000	-0.676527	-2.846194
6	0.000000	0.676527	-2.846194
1	0.000000	1.374576	-3.666455
1	-0.000000	-1.374576	-3.666455
6	-0.000000	0.000000	3.983971
35	-0.000000	0.000000	2.155242
6	0.000000	2.455656	-1.056714
6	-1.267201	3.170435	-1.514570
6	1.267201	3.170435	-1.514570
1	-0.000000	2.380169	0.031817
1	-2.154859	2.631543	-1.180036
1	-1.295146	4.180961	-1.102891
1	-1.299405	3.250436	-2.604521
1	2.154859	2.631543	-1.180036
1	1.299405	3.250436	-2.604521
1	1.295146	4.180961	-1.102891
6	-0.000000	-2.455656	-1.056714
6	1.267201	-3.170435	-1.514570
6	-1.267201	-3.170435	-1.514570
1	-0.000000	-2.380169	0.031817
1	2.154859	-2.631543	-1.180036
1	1.295146	-4.180961	-1.102891
1	1.299405	-3.250436	-2.604521
1	-2.154859	-2.631543	-1.180036
1	-1.299405	-3.250436	-2.604521
1	-1.295146	-4.180961	-1.102891
7	-0.000000	0.000000	5.135722

IiPr2-ICN

7	1.696338	1.067897	0.081567
6	0.874410	-0.000107	0.179499
7	1.695770	-1.068567	0.081703
6	3.010014	-0.677743	-0.097150
6	3.010372	0.676354	-0.097250
1	3.825059	1.372973	-0.194367
1	3.824336	-1.374801	-0.194158
6	-3.802798	0.000665	0.008110
6	1.214757	2.459319	0.078022
6	1.070342	2.953634	-1.357233
6	2.123514	3.350121	0.915756
1	0.229624	2.412335	0.544487
1	0.390327	2.308261	-1.915787
1	0.671725	3.969748	-1.365666
1	2.041315	2.960066	-1.860035
1	2.263379	2.938594	1.916551
1	3.102439	3.477436	0.446787
1	1.673277	4.339548	1.007584
6	1.213404	-2.459723	0.078231
6	2.122215	-3.351206	0.915180

6	1.067752	-2.953692	-1.357019
1	0.228611	-2.412285	0.545367
1	2.263115	-2.939895	1.915919
1	1.671385	-4.340340	1.007236
1	3.100693	-3.479134	0.445447
1	0.387692	-2.307860	-1.914987
1	2.038386	-2.960527	-1.860470
1	0.668597	-3.969597	-1.365373
7	-4.955092	0.000840	-0.058071
53	-1.676660	0.000350	0.122796

IiPr2-ClCCH

7	0.000000	1.060914	-1.234423
6	-0.000000	0.000000	-0.383123
7	-0.000000	-1.060914	-1.234423
6	-0.000000	-0.676762	-2.564753
6	0.000000	0.676762	-2.564753
1	0.000000	1.374279	-3.385801
1	-0.000000	-1.374279	-3.385801
6	0.000000	0.000000	4.327410
6	0.000000	0.000000	5.526704
1	0.000000	0.000000	6.589629
17	0.000000	0.000000	2.676532
6	-0.000000	-2.450217	-0.770623
6	1.266845	-3.168680	-1.225200
6	-1.266845	-3.168680	-1.225200
1	-0.000000	-2.366889	0.317517
1	2.153883	-2.628901	-0.890361
1	1.293464	-4.178750	-0.811707
1	1.300853	-3.250261	-2.315138
1	-2.153883	-2.628901	-0.890361
1	-1.300853	-3.250261	-2.315138
1	-1.293464	-4.178750	-0.811707
6	0.000000	2.450217	-0.770623
6	-1.266845	3.168680	-1.225200
6	1.266845	3.168680	-1.225200
1	0.000000	2.366889	0.317517
1	-2.153883	2.628901	-0.890361
1	-1.293464	4.178750	-0.811707
1	-1.300853	3.250261	-2.315138
1	2.153883	2.628901	-0.890361
1	1.300853	3.250261	-2.315138
1	1.293464	4.178750	-0.811707

IiPr2-BrCCH

7	0.000000	1.061975	-1.590764
6	0.000000	-0.000000	-0.742648
7	-0.000000	-1.061975	-1.590764
6	-0.000000	-0.676564	-2.920461
6	0.000000	0.676564	-2.920461
1	0.000000	1.374188	-3.741261
1	-0.000000	-1.374188	-3.741261

6	-0.000000	0.000000	4.066550
6	-0.000000	0.000000	5.267744
1	-0.000000	0.000000	6.331202
35	-0.000000	0.000000	2.253382
6	0.000000	2.452143	-1.127212
6	-1.266960	3.169392	-1.582725
6	1.266960	3.169392	-1.582725
1	0.000000	2.371176	-0.038967
1	-2.154042	2.629449	-1.248319
1	-1.294570	4.179351	-1.169216
1	-1.300125	3.251007	-2.672665
1	2.154042	2.629449	-1.248319
1	1.300125	3.251007	-2.672665
1	1.294570	4.179351	-1.169216
6	-0.000000	-2.452143	-1.127212
6	1.266960	-3.169392	-1.582725
6	-1.266960	-3.169392	-1.582725
1	-0.000000	-2.371176	-0.038967
1	2.154042	-2.629449	-1.248319
1	1.294570	-4.179351	-1.169216
1	1.300125	-3.251007	-2.672665
1	-2.154042	-2.629449	-1.248319
1	-1.300125	-3.251007	-2.672665
1	-1.294570	-4.179351	-1.169216

IiPr2-ICCH

7	1.812864	1.063948	-0.052095
6	0.984749	-0.000013	-0.195970
7	1.812283	-1.064393	-0.051753
6	3.118182	-0.677333	0.192237
6	3.118554	0.676259	0.192046
1	3.926605	1.373512	0.335255
1	3.925865	-1.374995	0.335557
6	-3.879351	0.000436	0.033550
6	-5.081537	0.000527	0.119004
1	-6.143136	0.000606	0.193741
53	-1.840823	0.000277	-0.109285
6	1.341601	2.453747	-0.087214
6	2.181525	3.286653	-1.048902
6	1.318038	3.035911	1.322345
1	0.320511	2.384574	-0.465867
1	2.210562	2.826096	-2.037553
1	1.751620	4.285396	-1.141908
1	3.206517	3.399588	-0.685980
1	0.692024	2.424727	1.974535
1	2.327310	3.073716	1.741764
1	0.917526	4.051324	1.303962
6	1.340421	-2.453979	-0.086932
6	1.317782	-3.036628	1.322437
6	2.179252	-3.286882	-1.049587
1	0.319059	-2.384277	-0.464740
1	0.692551	-2.425441	1.975375
1	0.916853	-4.051874	1.304015
1	2.327372	-3.075010	1.741040

1	2.207616	-2.826012	-2.038109
1	3.204513	-3.400243	-0.687553
1	1.748938	-4.285454	-1.142545

ItBu2-ClCN

7	-0.000000	1.065163	-1.285219
6	0.000000	0.000000	-0.431785
7	-0.000000	-1.065163	-1.285219
6	0.000000	-0.674066	-2.614804
6	0.000000	0.674066	-2.614804
1	-0.000000	1.365196	-3.440058
1	-0.000000	-1.365196	-3.440058
6	-0.000000	-0.000000	4.356394
17	-0.000000	-0.000000	2.712509
6	0.000000	-2.495908	-0.898550
6	-1.259993	-3.154447	-1.469498
6	1.259993	-3.154447	-1.469498
6	0.000000	-2.626256	0.618514
1	-2.152054	-2.657295	-1.082915
1	-1.282419	-3.105981	-2.560274
1	-1.292011	-4.206579	-1.178431
1	2.152054	-2.657295	-1.082915
1	1.292011	-4.206579	-1.178431
1	1.282419	-3.105981	-2.560274
1	0.000000	-3.687683	0.876313
1	0.883905	-2.157113	1.050792
1	-0.883905	-2.157113	1.050792
6	0.000000	2.495908	-0.898550
6	-1.259993	3.154447	-1.469498
6	0.000000	2.626256	0.618514
6	1.259993	3.154447	-1.469498
1	-1.282419	3.105981	-2.560274
1	-2.152054	2.657295	-1.082915
1	-1.292011	4.206579	-1.178431
1	0.883905	2.157113	1.050792
1	0.000000	3.687683	0.876313
1	-0.883905	2.157113	1.050792
1	1.292011	4.206579	-1.178431
1	2.152054	2.657295	-1.082915
1	1.282419	3.105981	-2.560274
7	-0.000000	-0.000000	5.507082

ItBu2-BrCN

7	0.000000	1.066310	-1.619288
6	0.000000	0.000000	-0.765116
7	-0.000000	-1.066310	-1.619288
6	0.000000	-0.673510	-2.947842
6	0.000000	0.673510	-2.947842
1	0.000000	1.364298	-3.773061
1	-0.000000	-1.364298	-3.773061
6	-0.000000	-0.000000	4.189830
35	-0.000000	-0.000000	2.383177

6	0.000000	2.503788	-1.252109
6	1.260378	3.153239	-1.833117
6	-1.260378	3.153239	-1.833117
6	0.000000	2.662503	0.260669
1	2.152359	2.660767	-1.440438
1	1.282844	3.091768	-2.923084
1	1.292960	4.208783	-1.555011
1	-2.152359	2.660767	-1.440438
1	-1.292960	4.208783	-1.555011
1	-1.282844	3.091768	-2.923084
1	0.000000	3.728458	0.498456
1	-0.884926	2.204910	0.702766
1	0.884926	2.204910	0.702766
6	-0.000000	-2.503788	-1.252109
6	1.260378	-3.153239	-1.833117
6	-0.000000	-2.662503	0.260669
6	-1.260378	-3.153239	-1.833117
1	1.282844	-3.091768	-2.923084
1	2.152359	-2.660767	-1.440438
1	1.292960	-4.208783	-1.555011
1	-0.884926	-2.204910	0.702766
1	-0.000000	-3.728458	0.498456
1	0.884926	-2.204910	0.702766
1	-1.292960	-4.208783	-1.555011
1	-2.152359	-2.660767	-1.440438
1	-1.282844	-3.091768	-2.923084
7	-0.000000	-0.000000	5.341288

ItBu2-ICN

7	0.000000	1.068164	-1.884912
6	-0.000000	-0.000000	-1.029785
7	-0.000000	-1.068164	-1.884912
6	-0.000000	-0.672683	-3.211975
6	0.000000	0.672683	-3.211975
1	0.000000	1.362672	-4.037376
1	-0.000000	-1.362672	-4.037376
6	0.000000	0.000000	4.156135
53	0.000000	0.000000	2.132201
6	0.000000	2.515742	-1.546940
6	1.261070	3.151079	-2.142683
6	-1.261070	3.151079	-2.142683
6	0.000000	2.717889	-0.041311
1	2.152871	2.666392	-1.740058
1	1.284235	3.069361	-3.231063
1	1.294077	4.211645	-1.884815
1	-2.152871	2.666392	-1.740058
1	-1.294077	4.211645	-1.884815
1	-1.284235	3.069361	-3.231063
1	0.000000	3.790350	0.164446
1	-0.886637	2.278420	0.415079
1	0.886637	2.278420	0.415079
6	-0.000000	-2.515742	-1.546940
6	1.261070	-3.151079	-2.142683
6	-0.000000	-2.717889	-0.041311

6	-1.261070	-3.151079	-2.142683
1	1.284235	-3.069361	-3.231063
1	2.152871	-2.666392	-1.740058
1	1.294077	-4.211645	-1.884815
1	-0.886637	-2.278420	0.415079
1	-0.000000	-3.790350	0.164446
1	0.886637	-2.278420	0.415079
1	-1.294077	-4.211645	-1.884815
1	-2.152871	-2.666392	-1.740058
1	-1.284235	-3.069361	-3.231063
7	0.000000	0.000000	5.308626

ItBu2-ClCCH

7	-0.000000	1.064454	-1.335219
6	0.000000	0.000000	-0.481796
7	-0.000000	-1.064454	-1.335219
6	0.000000	-0.674412	-2.665487
6	0.000000	0.674412	-2.665487
1	-0.000000	1.365538	-3.490966
1	-0.000000	-1.365538	-3.490966
6	0.000000	0.000000	4.445024
6	0.000000	0.000000	5.643901
1	0.000000	0.000000	6.706883
17	0.000000	0.000000	2.797553
6	0.000000	-2.491074	-0.937025
6	-1.259665	-3.154723	-1.502667
6	1.259665	-3.154723	-1.502667
6	0.000000	-2.604896	0.582019
1	-2.151450	-2.654620	-1.119284
1	-1.282213	-3.113853	-2.593935
1	-1.291530	-4.204768	-1.203800
1	2.151450	-2.654620	-1.119284
1	1.291530	-4.204768	-1.203800
1	1.282213	-3.113853	-2.593935
1	0.000000	-3.663732	0.851306
1	0.881434	-2.127885	1.010582
1	-0.881434	-2.127885	1.010582
6	0.000000	2.491074	-0.937025
6	-1.259665	3.154723	-1.502667
6	0.000000	2.604896	0.582019
6	1.259665	3.154723	-1.502667
1	-1.282213	3.113853	-2.593935
1	-2.151450	2.654620	-1.119284
1	-1.291530	4.204768	-1.203800
1	0.881434	2.127885	1.010582
1	0.000000	3.663732	0.851306
1	-0.881434	2.127885	1.010582
1	1.291530	4.204768	-1.203800
1	2.151450	2.654620	-1.119284
1	1.282213	3.113853	-2.593935

ItBu2-BrCCH

7	0.000000	1.065147	-1.693941
6	0.000000	0.000000	-0.839647
7	-0.000000	-1.065147	-1.693941
6	-0.000000	-0.673978	-3.023521
6	0.000000	0.673978	-3.023521
1	0.000000	1.365002	-3.848847
1	-0.000000	-1.365002	-3.848847
6	-0.000000	-0.000000	4.281831
6	-0.000000	-0.000000	5.482308
1	-0.000000	-0.000000	6.545768
35	-0.000000	-0.000000	2.479539
6	0.000000	2.496640	-1.309930
6	1.259960	3.154078	-1.882576
6	-1.259960	3.154078	-1.882576
6	0.000000	2.630210	0.206352
1	2.151734	2.657194	-1.494991
1	1.282527	3.104091	-2.973344
1	1.292241	4.206617	-1.592796
1	-2.151734	2.657194	-1.494991
1	-1.292241	4.206617	-1.592796
1	-1.282527	3.104091	-2.973344
1	0.000000	3.692322	0.462020
1	-0.882988	2.161760	0.640944
1	0.882988	2.161760	0.640944
6	-0.000000	-2.496640	-1.309930
6	1.259960	-3.154078	-1.882576
6	-0.000000	-2.630210	0.206352
6	-1.259960	-3.154078	-1.882576
1	1.282527	-3.104091	-2.973344
1	2.151734	-2.657194	-1.494991
1	1.292241	-4.206617	-1.592796
1	-0.882988	-2.161760	0.640944
1	-0.000000	-3.692322	0.462020
1	0.882988	-2.161760	0.640944
1	-1.292241	-4.206617	-1.592796
1	-2.151734	-2.657194	-1.494991
1	-1.282527	-3.104091	-2.973344

ItBu2-ICCH

7	-0.000000	1.066165	-1.988967
6	0.000000	-0.000000	-1.133291
7	-0.000000	-1.066165	-1.988967
6	0.000000	-0.673366	-3.317556
6	0.000000	0.673366	-3.317556
1	-0.000000	1.364055	-4.142838
1	-0.000000	-1.364055	-4.142838
6	0.000000	0.000000	4.242461
6	0.000000	0.000000	5.445740
1	0.000000	0.000000	6.509835
53	0.000000	0.000000	2.238526
6	-0.000000	2.504755	-1.626065
6	1.260384	3.152501	-2.209295

6	-1.260384	3.152501	-2.209295
6	-0.000000	2.668403	-0.114505
1	2.152073	2.660888	-1.814893
1	1.283158	3.088313	-3.299149
1	1.292993	4.208730	-1.933619
1	-2.152073	2.660888	-1.814893
1	-1.292993	4.208730	-1.933619
1	-1.283158	3.088313	-3.299149
1	-0.000000	3.735127	0.120515
1	-0.884150	2.211804	0.330061
1	0.884150	2.211804	0.330061
6	-0.000000	-2.504755	-1.626065
6	1.260384	-3.152501	-2.209295
6	-0.000000	-2.668403	-0.114505
6	-1.260384	-3.152501	-2.209295
1	1.283158	-3.088313	-3.299149
1	2.152073	-2.660888	-1.814893
1	1.292993	-4.208730	-1.933619
1	-0.884150	-2.211804	0.330061
1	-0.000000	-3.735127	0.120515
1	0.884150	-2.211804	0.330061
1	-1.292993	-4.208730	-1.933619
1	-2.152073	-2.660888	-1.814893
1	-1.283158	-3.088313	-3.299149

IPh2-ClCN

7	-1.376718	0.086386	1.060947
6	-0.525360	0.167783	-0.000000
7	-1.376718	0.086386	-1.060947
6	-2.706630	-0.041020	-0.673994
6	-2.706630	-0.041020	0.673994
1	-3.520875	-0.073969	1.376992
1	-3.520875	-0.073969	-1.376992
6	-0.934596	0.103275	2.415255
6	-1.590910	-0.667159	3.369825
6	0.157648	0.889983	2.769522
6	-1.156310	-0.639452	4.690522
1	-2.417604	-1.304031	3.078386
6	0.592421	0.898765	4.088236
1	0.649771	1.480662	2.007686
6	-0.064308	0.140376	5.053395
1	-1.666090	-1.241856	5.432607
1	1.444234	1.508656	4.363919
1	0.276264	0.154781	6.081344
6	-0.934596	0.103275	-2.415255
6	-1.590910	-0.667159	-3.369825
6	0.157648	0.889983	-2.769522
6	-1.156310	-0.639452	-4.690522
1	-2.417604	-1.304031	-3.078386
6	0.592421	0.898765	-4.088236
1	0.649771	1.480662	-2.007686
6	-0.064308	0.140376	-5.053395
1	-1.666090	-1.241856	-5.432607
1	1.444234	1.508656	-4.363919

1	0.276264	0.154781	-6.081344
6	4.034808	-0.481555	0.000000
17	2.406097	-0.238599	0.000000
7	5.173507	-0.648042	0.000000

IPh2-BrCN

7	-1.629737	0.083858	1.061600
6	-0.783377	0.181979	-0.000000
7	-1.629737	0.083858	-1.061600
6	-2.955832	-0.071908	-0.674188
6	-2.955832	-0.071908	0.674188
1	-3.767407	-0.123349	1.379221
1	-3.767407	-0.123349	-1.379221
6	-1.190571	0.112656	2.417674
6	-1.800655	-0.709169	3.359701
6	-0.150815	0.961765	2.783382
6	-1.370802	-0.671625	4.681500
1	-2.586273	-1.391050	3.056335
6	0.281064	0.979949	4.103162
1	0.304721	1.593780	2.031844
6	-0.329162	0.169531	5.056142
1	-1.843014	-1.313951	5.414837
1	1.093416	1.637187	4.388584
1	0.008710	0.191155	6.084854
6	-1.190571	0.112656	-2.417674
6	-1.800655	-0.709169	-3.359701
6	-0.150815	0.961765	-2.783382
6	-1.370802	-0.671625	-4.681500
1	-2.586273	-1.391050	-3.056335
6	0.281064	0.979949	-4.103162
1	0.304721	1.593780	-2.031844
6	-0.329162	0.169531	-5.056142
1	-1.843014	-1.313951	-5.414837
1	1.093416	1.637187	-4.388584
1	0.008710	0.191155	-6.084854
6	3.881531	-0.424864	0.000000
35	2.081399	-0.174432	0.000000
7	5.022769	-0.579073	0.000000

IPh2-ICN

7	-1.063643	-1.777085	-0.065620
6	0.000095	-0.942550	-0.189859
7	1.063900	-1.776995	-0.065632
6	0.674809	-3.094757	0.136718
6	-0.674437	-3.094807	0.136771
1	-1.382823	-3.901040	0.218699
1	1.383266	-3.900931	0.218614
6	-2.423767	-1.344120	-0.115027
6	-3.341596	-1.861332	0.792373
6	-2.810496	-0.409506	-1.068990
6	-4.665430	-1.440623	0.736977
1	-3.016372	-2.565683	1.549091

6	-4.132189	0.015997	-1.105088
1	-2.076493	-0.025420	-1.766163
6	-5.062042	-0.500452	-0.207932
1	-5.382517	-1.837558	1.445030
1	-4.435863	0.749803	-1.841458
1	-6.092037	-0.167644	-0.242573
6	2.423986	-1.343896	-0.115020
6	3.341868	-1.861073	0.792344
6	2.810620	-0.409198	-1.068940
6	4.665663	-1.440238	0.736962
1	3.016716	-2.565499	1.549023
6	4.132273	0.016429	-1.105024
1	2.076577	-0.025148	-1.766090
6	5.062179	-0.499980	-0.207899
1	5.382792	-1.837146	1.444986
1	4.435875	0.750301	-1.841358
1	6.092143	-0.167074	-0.242529
6	-0.000385	3.792526	0.437675
53	-0.000116	1.742419	0.120664
7	-0.000543	4.932692	0.611723

IPh2-ClCCH

7	-1.410113	0.092500	1.060689
6	-0.554049	0.137353	0.000000
7	-1.410113	0.092500	-1.060689
6	-2.744887	0.024996	-0.673799
6	-2.744887	0.024996	0.673799
1	-3.560913	0.028983	1.375387
1	-3.560913	0.028983	-1.375387
6	-0.969109	0.092215	2.414978
6	-1.680460	-0.616988	3.377980
6	0.177693	0.800775	2.762654
6	-1.247951	-0.603881	4.699661
1	-2.550151	-1.197046	3.093446
6	0.608502	0.795099	4.082481
1	0.716051	1.339405	1.993814
6	-0.103610	0.099931	5.056192
1	-1.801616	-1.158585	5.447568
1	1.502845	1.343874	4.351976
1	0.234711	0.103434	6.085045
6	-0.969109	0.092215	-2.414978
6	-1.680460	-0.616988	-3.377980
6	0.177693	0.800775	-2.762654
6	-1.247951	-0.603881	-4.699661
1	-2.550151	-1.197046	-3.093446
6	0.608502	0.795099	-4.082481
1	0.716051	1.339405	-1.993814
6	-0.103610	0.099931	-5.056192
1	-1.801616	-1.158585	-5.447568
1	1.502845	1.343874	-4.351976
1	0.234711	0.103434	-6.085045
6	4.132525	-0.405537	-0.000000
6	5.325206	-0.529286	-0.000000
1	6.382014	-0.643362	-0.000000
17	2.492557	-0.228983	-0.000000

IPh2-BrCCH

7	-1.063592	-1.683824	-0.085242
6	-0.001705	-0.833245	-0.166378
7	1.058501	-1.685918	-0.085375
6	0.670078	-3.015493	0.040808
6	-0.677759	-3.014165	0.040883
1	-1.381588	-3.827667	0.073110
1	1.372335	-3.830358	0.072973
6	-2.418634	-1.243712	-0.104296
6	-3.373107	-1.903728	0.663352
6	-2.773772	-0.151085	-0.889699
6	-4.695038	-1.473329	0.633044
1	-3.080653	-2.730133	1.300136
6	-4.093686	0.279839	-0.900403
1	-2.012076	0.347213	-1.475151
6	-5.059040	-0.381110	-0.146050
1	-5.437009	-1.986113	1.233092
1	-4.369135	1.133511	-1.507743
1	-6.087969	-0.043410	-0.162306
6	2.414378	-1.248402	-0.104544
6	3.367518	-1.909822	0.663543
6	2.771620	-0.156838	-0.890464
6	4.690241	-1.481867	0.633208
1	3.073419	-2.735382	1.300671
6	4.092321	0.271669	-0.901185
1	2.010929	0.342484	-1.476365
6	5.056341	-0.390679	-0.146350
1	5.431197	-1.995718	1.233597
1	4.369435	1.124526	-1.508914
1	6.085890	-0.054875	-0.162615
6	0.006966	3.984063	0.369532
6	0.009040	5.177718	0.501153
1	0.010868	6.234433	0.620502
35	0.003869	2.187166	0.167897

IPh2-ICCH

7	-1.062507	-1.876525	-0.057368
6	-0.000428	-1.036981	-0.198302
7	1.061264	-1.877000	-0.057400
6	0.673303	-3.192865	0.166501
6	-0.675121	-3.192565	0.166513
1	-1.380741	-3.999897	0.259694
1	1.378596	-4.000484	0.259693
6	-2.419463	-1.441388	-0.110771
6	-3.359678	-2.004352	0.745975
6	-2.786796	-0.453354	-1.018591
6	-4.682275	-1.579969	0.684170
1	-3.054047	-2.748115	1.472367
6	-4.107300	-0.025566	-1.060889
1	-2.035836	-0.030620	-1.673692
6	-5.058790	-0.589520	-0.216111
1	-5.414428	-2.014951	1.353600
1	-4.393541	0.748400	-1.762520

1	-6.088071	-0.254962	-0.256495
6	2.418424	-1.442499	-0.110839
6	3.358130	-2.005106	0.746698
6	2.786451	-0.455477	-1.019471
6	4.680925	-1.581344	0.684921
1	3.051916	-2.748099	1.473635
6	4.107153	-0.028282	-1.061743
1	2.035918	-0.033115	-1.675306
6	5.058127	-0.591853	-0.216134
1	5.412693	-2.016031	1.354961
1	4.393954	0.744903	-1.764006
1	6.087560	-0.257760	-0.256494
6	0.001465	3.900288	0.400083
6	0.001928	5.092934	0.567623
1	0.002356	6.146585	0.716461
53	0.000680	1.896344	0.120824

IMes2-ClCN

7	1.579124	-0.880267	0.398988
6	0.398046	-0.275236	0.086214
7	-0.509660	-1.208994	0.493664
6	0.078624	-2.342160	1.037526
6	1.412283	-2.132888	0.976682
1	2.245282	-2.741414	1.286982
1	-0.498200	-3.172375	1.409827
6	2.860457	-0.287186	0.153516
6	3.603221	-0.720390	-0.945524
6	3.325003	0.701256	1.022968
6	4.853689	-0.143086	-1.157547
6	4.579672	1.251713	0.772109
6	5.356156	0.842427	-0.310779
1	5.444870	-0.466995	-2.008737
1	4.959509	2.019267	1.439698
6	-1.921911	-0.995454	0.367292
6	-2.537952	-1.281671	-0.850570
6	-2.606954	-0.399658	1.427044
6	-3.882714	-0.946252	-0.996017
6	-3.949678	-0.080945	1.239241
6	-4.599500	-0.334356	0.031282
1	-4.375766	-1.145528	-1.942604
1	-4.495055	0.400913	2.044983
6	6.695489	1.479228	-0.578632
1	7.356960	0.797030	-1.114432
1	6.577310	2.377982	-1.190196
1	7.184675	1.773315	0.351259
6	-6.023700	0.104022	-0.179095
1	-6.590962	0.065792	0.752109
1	-6.040004	1.135899	-0.543000
1	-6.525682	-0.522780	-0.917486
6	2.481567	1.161293	2.181697
1	1.593926	1.685123	1.818492
1	2.132330	0.313392	2.776288
1	3.047831	1.832439	2.827457
6	-1.883268	-0.044713	2.698196

1	-1.412409	-0.921020	3.149971
1	-1.086811	0.675459	2.488566
1	-2.569101	0.393164	3.423143
6	3.053533	-1.766801	-1.878680
1	3.005678	-2.746575	-1.396245
1	2.037574	-1.509421	-2.187531
1	3.679489	-1.855394	-2.766517
6	-1.743851	-1.856833	-1.992283
1	-1.020306	-1.119341	-2.351985
1	-1.177957	-2.738252	-1.682167
1	-2.399781	-2.134053	-2.817392
6	-3.133727	2.603736	-1.009224
7	-4.198590	3.018279	-1.150352
17	-1.626848	1.993175	-0.801122

IMes2-BrCN

7	1.358003	-1.295003	0.288448
6	0.222141	-0.581956	0.064894
7	-0.752836	-1.497341	0.306018
6	-0.246229	-2.736995	0.671667
6	1.099234	-2.607736	0.660465
1	1.885454	-3.308819	0.885200
1	-0.881994	-3.574748	0.904927
6	2.675194	-0.740504	0.172482
6	3.461902	-1.094320	-0.924243
6	3.125526	0.136303	1.162299
6	4.743344	-0.553048	-1.009314
6	4.412028	0.653462	1.034449
6	5.233257	0.320854	-0.041831
1	5.368549	-0.814508	-1.857519
1	4.781926	1.333798	1.795574
6	-2.150742	-1.185681	0.216201
6	-2.820776	-1.431973	-0.981707
6	-2.781198	-0.615610	1.323207
6	-4.174076	-1.107478	-1.048249
6	-4.134164	-0.305934	1.211721
6	-4.844338	-0.544210	0.035895
1	-4.713984	-1.290501	-1.972143
1	-4.643616	0.139039	2.060960
6	6.608023	0.924024	-0.170679
1	7.255086	0.303203	-0.791587
1	6.550138	1.913329	-0.632642
1	7.078879	1.042317	0.806575
6	-6.297670	-0.161577	-0.071513
1	-6.808837	-0.283186	0.884678
1	-6.394626	0.886003	-0.369294
1	-6.811954	-0.769110	-0.817276
6	2.241179	0.518816	2.319155
1	1.438313	1.179878	1.982521
1	1.768922	-0.360069	2.764775
1	2.817579	1.034648	3.087109
6	-2.003487	-0.316258	2.576678
1	-1.464405	-1.197896	2.931938
1	-1.258740	0.460981	2.382393

1	-2.667369	0.029571	3.368670
6	2.931389	-2.017165	-1.990061
1	2.866421	-3.047960	-1.631773
1	1.926673	-1.716763	-2.296328
1	3.582294	-2.004476	-2.864108
6	-2.084085	-1.995481	-2.167419
1	-1.279067	-1.319449	-2.468068
1	-1.625958	-2.959486	-1.932992
1	-2.759416	-2.130156	-3.011980
6	-1.276790	3.754753	-0.698418
7	-1.688528	4.818800	-0.856233
35	-0.630420	2.068442	-0.441466

IMes2-ICN

7	1.064634	-1.580993	0.146667
6	-0.000239	-0.753480	0.053384
7	-1.065251	-1.580824	0.146652
6	-0.676950	-2.903648	0.298944
6	0.676124	-2.903755	0.298968
1	1.391566	-3.703161	0.394982
1	-1.392522	-3.702940	0.394940
6	2.427409	-1.125284	0.111863
6	3.114255	-1.160809	-1.101633
6	2.999344	-0.640821	1.288123
6	4.431864	-0.711100	-1.111603
6	4.319399	-0.200460	1.229635
6	5.048085	-0.228575	0.042044
1	4.986047	-0.727833	-2.044857
1	4.786099	0.180965	2.132398
6	-2.427942	-1.124859	0.111810
6	-3.114803	-1.160412	-1.101653
6	-2.999793	-0.640196	1.288049
6	-4.432366	-0.710501	-1.111655
6	-4.319753	-0.199609	1.229522
6	-5.048475	-0.227737	0.041929
1	-4.986559	-0.727263	-2.044899
1	-4.786377	0.182007	2.132247
6	6.460735	0.292465	-0.004169
1	7.047441	-0.224917	-0.764457
1	6.464301	1.358257	-0.248081
1	6.958182	0.169982	0.958798
6	-6.461018	0.293606	-0.004173
1	-6.959555	0.167982	0.957836
1	-6.464184	1.360215	-0.244495
1	-7.046925	-0.221174	-0.766825
6	2.198183	-0.564737	2.560153
1	1.424751	0.204303	2.473179
1	1.695860	-1.511052	2.774833
1	2.839961	-0.311969	3.403720
6	-2.198636	-0.564231	2.560089
1	-1.696740	-1.510736	2.774935
1	-1.424861	0.204452	2.473042
1	-2.840341	-0.311052	3.403587
6	2.430887	-1.634852	-2.356539

1	2.080319	-2.665326	-2.259329
1	1.557729	-1.012534	-2.571278
1	3.109978	-1.581756	-3.206893
6	-2.431565	-1.634814	-2.356490
1	-1.557573	-1.013497	-2.570717
1	-2.082292	-2.665755	-2.259491
1	-3.110301	-1.580633	-3.207056
6	0.001191	3.894698	-0.379168
53	0.000443	1.775789	-0.194819
7	0.001603	5.044857	-0.476931

IMes2-ClCCH

7	0.454462	-1.228771	0.459379
6	-0.441969	-0.274911	0.073653
7	-1.629717	-0.876601	0.373365
6	-1.476709	-2.144522	0.921246
6	-0.145464	-2.368805	0.976659
1	0.422783	-3.213509	1.329042
1	-2.315910	-2.751774	1.217087
6	1.869451	-1.035033	0.339784
6	2.480484	-1.287695	-0.887576
6	2.565434	-0.495948	1.422224
6	3.834203	-0.984001	-1.016527
6	3.916791	-0.207935	1.250760
6	4.563271	-0.438010	0.037432
1	4.321823	-1.149135	-1.972508
1	4.469006	0.238221	2.072336
6	-2.904951	-0.267103	0.139688
6	-3.650940	-0.671046	-0.968515
6	-3.356794	0.715878	1.021780
6	-4.893456	-0.074553	-1.173235
6	-4.603861	1.286785	0.777695
6	-5.386947	0.899899	-0.308421
1	-5.483482	-0.370864	-2.035274
1	-4.970329	2.055480	1.451431
6	6.019033	-0.091751	-0.131881
1	6.245115	0.141755	-1.173521
1	6.284930	0.772848	0.478819
1	6.656487	-0.926826	0.172325
6	-6.747455	1.509060	-0.530454
1	-7.513934	0.945884	0.009258
1	-6.780387	2.539622	-0.173761
1	-7.013927	1.502970	-1.588368
6	1.843235	-0.155796	2.697971
1	1.077372	0.600499	2.502360
1	1.335203	-1.027355	3.117470
1	2.537544	0.233253	3.442581
6	-2.503888	1.155398	2.181393
1	-2.161266	0.297370	2.765244
1	-1.612134	1.672647	1.818985
1	-3.060664	1.825535	2.836512
6	1.672617	-1.783874	-2.055993
1	1.060311	-2.646061	-1.782020
1	0.991363	-0.997983	-2.394854

1	2.323541	-2.064352	-2.884138
6	-3.107119	-1.699891	-1.924400
1	-2.089688	-1.440865	-2.227209
1	-3.064210	-2.690161	-1.463423
1	-3.733745	-1.765599	-2.813824
6	3.336697	2.674846	-0.923245
6	4.449710	3.101662	-1.056345
1	5.432908	3.486707	-1.178048
17	1.816461	2.076081	-0.737521

IMes2-BrCCH

7	1.487017	-1.236889	0.334014
6	0.324365	-0.588361	0.045091
7	-0.611083	-1.525684	0.359887
6	-0.056905	-2.708996	0.829979
6	1.281659	-2.524557	0.813334
1	2.094412	-3.171462	1.098296
1	-0.658619	-3.551052	1.129056
6	2.781408	-0.642804	0.177017
6	3.595588	-1.065538	-0.874082
6	3.185474	0.341480	1.082768
6	4.856789	-0.484715	-0.998526
6	4.452375	0.894859	0.918199
6	5.300197	0.494716	-0.113825
1	5.502068	-0.799869	-1.812854
1	4.785558	1.658850	1.614318
6	-2.019836	-1.284949	0.239225
6	-2.652592	-1.575823	-0.968829
6	-2.699460	-0.730298	1.324255
6	-4.017446	-1.314662	-1.067960
6	-4.062841	-0.485489	1.181806
6	-4.735742	-0.770140	-0.005256
1	-4.528554	-1.533403	-2.000567
1	-4.609377	-0.051762	2.013672
6	6.652848	1.136928	-0.283999
1	7.317405	0.507162	-0.876791
1	6.559121	2.099027	-0.794967
1	7.125021	1.320851	0.682650
6	-6.202252	-0.455125	-0.149506
1	-6.723016	-0.556424	0.803887
1	-6.338707	0.573105	-0.495611
1	-6.678774	-1.116604	-0.874413
6	2.271329	0.795993	2.189066
1	1.439723	1.375811	1.780401
1	1.838843	-0.056246	2.719096
1	2.813760	1.415439	2.903493
6	-1.958853	-0.368627	2.583478
1	-1.409554	-1.223744	2.984882
1	-1.226903	0.417056	2.374059
1	-2.648454	-0.007862	3.346431
6	3.113551	-2.101934	-1.855327
1	3.089027	-3.098593	-1.406782
1	2.099375	-1.872683	-2.190632
1	3.770613	-2.139153	-2.724205

6	-1.864110	-2.113786	-2.132515
1	-1.122526	-1.377757	-2.454833
1	-1.320764	-3.022068	-1.860709
1	-2.521068	-2.339401	-2.972365
6	-1.787079	3.670926	-0.647547
6	-2.359411	4.719609	-0.772289
1	-2.865524	5.648121	-0.883950
35	-0.923113	2.091669	-0.458463

IMes2-ICCH

7	-1.228748	1.580840	0.221715
6	-0.124041	0.807098	0.064589
7	0.890527	1.694823	0.221834
6	0.436372	2.982019	0.473703
6	-0.913798	2.909317	0.473581
1	-1.669354	3.660308	0.632899
1	1.107655	3.809587	0.631436
6	-2.566113	1.065864	0.156988
6	-3.319988	1.294160	-0.995020
6	-3.061684	0.345285	1.244989
6	-4.617482	0.789255	-1.034361
6	-4.363737	-0.142548	1.159672
6	-5.153543	0.068921	0.031353
1	-5.219067	0.954085	-1.922997
1	-4.769019	-0.704249	1.995716
6	2.274347	1.318322	0.161293
6	2.959276	1.463720	-1.044910
6	2.874848	0.792896	1.305901
6	4.299529	1.086326	-1.080209
6	4.216247	0.427580	1.224349
6	4.941790	0.567523	0.042528
1	4.850865	1.190205	-2.009654
1	4.703286	0.016340	2.103189
6	-6.545142	-0.503126	-0.049863
1	-7.183472	0.101415	-0.695740
1	-6.517985	-1.515896	-0.461159
1	-7.006768	-0.558113	0.937107
6	6.380563	0.126029	-0.030499
1	6.882622	0.257794	0.929121
1	6.440755	-0.933257	-0.294784
1	6.928725	0.689595	-0.786753
6	-2.207152	0.087918	2.457259
1	-1.433507	-0.649572	2.225505
1	-1.700079	0.997835	2.787078
1	-2.813610	-0.293143	3.278918
6	2.079279	0.596453	2.568291
1	1.540965	1.505595	2.847024
1	1.334408	-0.191706	2.423500
1	2.731207	0.309458	3.393164
6	-2.732481	2.041986	-2.162753
1	-2.568266	3.095686	-1.922798
1	-1.764414	1.618594	-2.441624
1	-3.397866	1.989680	-3.024235
6	2.249561	1.975351	-2.269884

1	1.416866	1.314726	-2.526367
1	1.833673	2.972491	-2.105839
1	2.931416	2.021086	-3.118701
6	0.587251	-3.972383	-0.475914
6	0.779776	-5.156973	-0.587700
1	0.949032	-6.202794	-0.687647
53	0.262593	-1.966252	-0.284745

IDipp2-ClCN

7	0.677358	0.213833	-1.170041
6	-0.241080	0.043487	-0.176285
7	-1.412561	0.046542	-0.871764
6	-1.229994	0.209789	-2.239028
6	0.103151	0.325809	-2.427995
1	0.688198	0.473133	-3.320528
1	-2.054429	0.229391	-2.932124
6	2.081715	0.334149	-0.898692
6	2.562639	1.557801	-0.422999
6	2.899884	-0.792479	-1.070813
6	3.914672	1.628922	-0.080164
6	4.242719	-0.670315	-0.720444
6	4.743582	0.526781	-0.219871
1	4.319460	2.558564	0.304353
1	4.907461	-1.517859	-0.831791
1	5.788486	0.597922	0.058548
6	-2.702540	-0.129420	-0.265767
6	-3.486991	1.007868	-0.022697
6	-3.124992	-1.425016	0.048342
6	-4.742706	0.813710	0.549088
6	-4.386438	-1.567930	0.630102
6	-5.187956	-0.462845	0.873016
1	-5.380515	1.665723	0.749108
1	-4.743549	-2.556180	0.895726
1	-6.166321	-0.594015	1.320563
6	2.323436	-2.110967	-1.562804
6	1.711324	-2.894169	-0.392558
6	3.345772	-2.974501	-2.304396
1	1.515789	-1.880894	-2.263377
1	0.926415	-2.319497	0.101725
1	1.281873	-3.834890	-0.749339
1	2.481134	-3.127638	0.349270
1	3.858425	-2.411693	-3.087616
1	4.098787	-3.377457	-1.622513
1	2.841116	-3.825524	-2.766614
6	-2.234966	-2.635513	-0.170212
6	-2.983112	-3.807205	-0.812873
6	-1.602440	-3.054022	1.163799
1	-1.426962	-2.346920	-0.847827
1	-3.479608	-3.504535	-1.737304
1	-2.280900	-4.610998	-1.046315
1	-3.738740	-4.220589	-0.140467
1	-1.029378	-2.229426	1.593202
1	-2.379054	-3.341671	1.878420
1	-0.934613	-3.907677	1.021455

6	-2.944948	2.400174	-0.300212
6	-2.082531	2.860842	0.884996
6	-4.035773	3.429305	-0.600570
1	-2.296265	2.340782	-1.178541
1	-1.277330	2.151265	1.087702
1	-1.642715	3.840508	0.677290
1	-2.698519	2.945536	1.784933
1	-4.712126	3.084041	-1.385722
1	-4.629320	3.655565	0.288769
1	-3.577830	4.364802	-0.928838
6	1.674950	2.780819	-0.265337
6	2.196806	3.949700	-1.109492
6	1.550843	3.180648	1.208990
1	0.674024	2.530590	-0.625396
1	2.286303	3.668335	-2.161089
1	1.514852	4.800452	-1.036699
1	3.179723	4.280225	-0.763942
1	1.120417	2.368213	1.796968
1	2.529172	3.426967	1.630854
1	0.907894	4.058310	1.311727
6	2.444789	-0.813385	3.525191
17	1.381044	-0.482560	2.315208
7	3.191206	-1.046315	4.369664

IDipp2-BrCN

7	0.788127	-0.037502	-1.351107
6	-0.180409	-0.059083	-0.396286
7	-1.320484	-0.037454	-1.135233
6	-1.073177	-0.005943	-2.500725
6	0.272087	0.003329	-2.637839
1	0.901530	0.031021	-3.511657
1	-1.866576	0.008456	-3.229175
6	2.186756	0.036254	-1.032523
6	2.732184	1.288459	-0.731707
6	2.935622	-1.148898	-1.015040
6	4.085124	1.334123	-0.389749
6	4.281301	-1.051639	-0.667063
6	4.849434	0.177963	-0.353246
1	4.543332	2.287035	-0.149775
1	4.895380	-1.943127	-0.638193
1	5.897022	0.232309	-0.081008
6	-2.637165	-0.059121	-0.562095
6	-3.308460	1.160167	-0.389365
6	-3.188212	-1.291224	-0.198463
6	-4.586039	1.116191	0.164830
6	-4.467624	-1.282944	0.361060
6	-5.159750	-0.094395	0.536992
1	-5.139715	2.034984	0.313555
1	-4.923751	-2.218094	0.665259
1	-6.152912	-0.107764	0.970649
6	2.284151	-2.489679	-1.312169
6	1.638473	-3.057960	-0.040167
6	3.253017	-3.506909	-1.918454
1	1.486330	-2.318038	-2.040702

1	0.885837	-2.377158	0.361491
1	1.160669	-4.018168	-0.254168
1	2.398956	-3.216517	0.729847
1	3.789889	-3.093272	-2.774979
1	3.987837	-3.846370	-1.184199
1	2.699999	-4.387616	-2.251700
6	-2.420763	-2.593277	-0.343795
6	-3.249289	-3.679428	-1.037002
6	-1.936323	-3.064283	1.033411
1	-1.537997	-2.404583	-0.960362
1	-3.621682	-3.338098	-2.005284
1	-2.636450	-4.569442	-1.197848
1	-4.107326	-3.978067	-0.429891
1	-1.316218	-2.301021	1.508416
1	-2.787516	-3.268188	1.689335
1	-1.349224	-3.981417	0.940637
6	-2.631138	2.478769	-0.724292
6	-1.792806	2.950689	0.473913
6	-3.613346	3.570840	-1.152525
1	-1.947541	2.303505	-1.559895
1	-1.051821	2.201319	0.761251
1	-1.271881	3.880830	0.230153
1	-2.440839	3.136577	1.335139
1	-4.277941	3.226596	-1.947999
1	-4.227742	3.908532	-0.314267
1	-3.061218	4.439441	-1.517632
6	1.918590	2.570057	-0.798425
6	2.393817	3.439572	-1.969657
6	1.974628	3.348283	0.520026
1	0.873347	2.307501	-0.978733
1	2.325806	2.898185	-2.916156
1	1.784320	4.343555	-2.044448
1	3.434698	3.744074	-1.830109
1	1.618792	2.737553	1.351745
1	2.992730	3.674426	0.747300
1	1.347359	4.240620	0.455318
6	1.298713	0.015986	4.039443
35	0.667487	-0.018803	2.330813
7	1.700369	0.039691	5.118497

IDipp2-ICN

7	1.063697	0.004470	-1.368962
6	0.000132	0.000466	-0.532430
7	-1.063413	-0.002767	-1.368971
6	-0.676452	-0.006651	-2.699985
6	0.676749	0.009506	-2.699973
1	1.392417	0.020163	-3.504898
1	-1.392115	-0.016608	-3.504922
6	2.425144	0.064591	-0.908855
6	2.945327	1.310273	-0.545244
6	3.155490	-1.129474	-0.828529
6	4.257629	1.339876	-0.070023
6	4.460701	-1.045922	-0.348531
6	5.004377	0.176144	0.031019

1	4.696712	2.286371	0.224563
1	5.061144	-1.943243	-0.265560
1	6.019841	0.218400	0.407082
6	-2.424908	-0.062794	-0.909009
6	-3.154584	1.131553	-0.826939
6	-2.945824	-1.308734	-0.547337
6	-4.459904	1.047994	-0.347214
6	-4.258205	-1.338350	-0.072366
6	-5.004316	-0.174345	0.030375
1	-5.059842	1.945533	-0.262927
1	-4.697856	-2.285046	0.220720
1	-6.019849	-0.216610	0.406249
6	2.528756	-2.463956	-1.200762
6	1.793754	-3.065053	0.006283
6	3.543582	-3.465212	-1.757703
1	1.786930	-2.279817	-1.983230
1	1.012630	-2.398549	0.377024
1	1.334597	-4.019009	-0.267605
1	2.495917	-3.244417	0.825133
1	4.140609	-3.030296	-2.562224
1	4.223378	-3.820721	-0.979604
1	3.019640	-4.338722	-2.151276
6	-2.153608	-2.597828	-0.683441
6	-2.760255	-3.481463	-1.780996
6	-2.068410	-3.353972	0.646568
1	-1.132844	-2.348217	-0.983227
1	-2.793621	-2.956689	-2.738716
1	-2.168485	-4.391513	-1.906005
1	-3.781086	-3.775505	-1.522572
1	-1.641999	-2.724960	1.430091
1	-3.055266	-3.688720	0.976422
1	-1.437928	-4.239206	0.533343
6	-2.527111	2.466258	-1.197086
6	-1.792871	3.065662	0.011264
6	-3.541193	3.468479	-1.753661
1	-1.784730	2.282813	-1.979191
1	-1.012299	2.398409	0.381837
1	-1.333130	4.019767	-0.261127
1	-2.495632	3.244338	0.829752
1	-4.137656	3.034688	-2.559205
1	-4.221559	3.823231	-0.975717
1	-3.016683	4.342322	-2.145736
6	2.152506	2.599207	-0.679431
6	2.759668	3.485500	-1.774552
6	2.065660	3.352465	0.652109
1	1.132155	2.349701	-0.980701
1	2.794271	2.962737	-2.733324
1	2.167450	4.395441	-1.898224
1	3.780064	3.779653	-1.514541
1	1.638633	2.721628	1.433838
1	3.052075	3.686780	0.983728
1	1.435017	4.237744	0.540156
6	-0.000296	-0.004757	4.175480
53	-0.000178	-0.002216	2.068603
7	-0.000382	-0.006157	5.329429

IDipp2-ClCCH

7	0.564747	0.333277	-1.159546
6	-0.316548	0.093831	-0.144749
7	-1.504018	0.044403	-0.814689
6	-1.365499	0.241240	-2.183119
6	-0.046375	0.434891	-2.401355
1	0.506754	0.630643	-3.304865
1	-2.206463	0.226884	-2.856249
6	1.969627	0.507460	-0.924715
6	2.406809	1.719712	-0.383672
6	2.833897	-0.567150	-1.183915
6	3.761404	1.831105	-0.062285
6	4.177208	-0.405483	-0.853448
6	4.635209	0.779198	-0.286991
1	4.131600	2.750521	0.377411
1	4.875476	-1.214633	-1.027650
1	5.681265	0.879293	-0.021783
6	-2.765454	-0.224414	-0.184415
6	-3.629056	0.851313	0.072159
6	-3.084906	-1.547299	0.138910
6	-4.855378	0.565606	0.668799
6	-4.320212	-1.782191	0.746418
6	-5.197197	-0.739616	1.004247
1	-5.551271	1.368050	0.879835
1	-4.597527	-2.793721	1.019484
1	-6.154052	-0.942155	1.471370
6	2.306245	-1.883869	-1.732327
6	1.763793	-2.753874	-0.589490
6	3.346491	-2.657988	-2.544385
1	1.469985	-1.658288	-2.399535
1	0.976500	-2.237535	-0.038163
1	1.359476	-3.690588	-0.984881
1	2.565795	-2.993936	0.115450
1	3.809640	-2.031663	-3.310169
1	4.136881	-3.058098	-1.904382
1	2.869455	-3.507908	-3.037201
6	-2.113861	-2.689498	-0.100993
6	-2.792592	-3.922586	-0.704785
6	-1.401655	-3.043797	1.211418
1	-1.356395	-2.348101	-0.811602
1	-3.351442	-3.668994	-1.608238
1	-2.037806	-4.668656	-0.964577
1	-3.482122	-4.390727	0.002020
1	-0.868563	-2.176421	1.606416
1	-2.128925	-3.373511	1.959294
1	-0.683765	-3.852775	1.051645
6	-3.197570	2.279437	-0.218059
6	-2.329992	2.797106	0.939267
6	-4.367605	3.229359	-0.477531
1	-2.575777	2.267929	-1.117298
1	-1.476624	2.139680	1.117478
1	-1.958878	3.802021	0.717575
1	-2.922236	2.844924	1.857775
1	-5.043250	2.839778	-1.242397
1	-4.946305	3.409873	0.431907

1	-3.987854	4.196063	-0.815157
6	1.463139	2.877391	-0.106268
6	1.954708	4.173156	-0.761300
6	1.273577	3.062894	1.403799
1	0.487581	2.636906	-0.536541
1	2.111374	4.040509	-1.834031
1	1.218940	4.967918	-0.615630
1	2.896409	4.510951	-0.321307
1	0.855323	2.160865	1.853647
1	2.230493	3.275197	1.889459
1	0.597125	3.898101	1.603356
6	3.114936	-1.043031	3.074529
6	4.087319	-1.377517	3.691239
1	4.949013	-1.671725	4.239574
17	1.783076	-0.590750	2.219748

IDipp2-BrCCH

7	-0.714039	-0.009421	-1.398856
6	0.225115	0.045928	-0.413850
7	1.385439	0.007921	-1.123927
6	1.178314	-0.066278	-2.494933
6	-0.162191	-0.086301	-2.669534
1	-0.766318	-0.143520	-3.559685
1	1.991898	-0.098985	-3.200201
6	-2.121110	-0.080059	-1.123267
6	-2.673683	-1.325245	-0.807801
6	-2.874779	1.101630	-1.163688
6	-4.038195	-1.367900	-0.514764
6	-4.232117	1.008258	-0.863670
6	-4.807781	-0.215040	-0.538871
1	-4.501065	-2.316121	-0.264959
1	-4.849520	1.897825	-0.880885
1	-5.864840	-0.266814	-0.305126
6	2.686373	0.047906	-0.518654
6	3.356002	-1.164568	-0.298424
6	3.226981	1.289607	-0.172042
6	4.623206	-1.103790	0.277712
6	4.495993	1.299080	0.410650
6	5.188362	0.117363	0.627581
1	5.174887	-2.017383	0.461959
1	4.943636	2.242541	0.701303
1	6.173928	0.144415	1.077794
6	-2.215502	2.436896	-1.467143
6	-1.617826	3.032320	-0.184189
6	-3.162543	3.438671	-2.130696
1	-1.390988	2.250732	-2.161564
1	-0.886949	2.356390	0.263250
1	-1.126965	3.985352	-0.401550
1	-2.407733	3.211448	0.551013
1	-3.665848	3.006146	-2.998323
1	-3.925422	3.791039	-1.431896
1	-2.599023	4.313984	-2.460804
6	2.450906	2.582179	-0.350261
6	3.290839	3.682595	-1.005633

6	1.903334	3.040923	1.007747
1	1.596963	2.381818	-1.002750
1	3.715226	3.347761	-1.954649
1	2.669043	4.560323	-1.197005
1	4.112806	3.999602	-0.359122
1	1.274030	2.267070	1.452747
1	2.725374	3.252357	1.697890
1	1.309083	3.951228	0.894170
6	2.681823	-2.492116	-0.601911
6	1.817065	-2.916749	0.595567
6	3.668172	-3.601966	-0.970357
1	2.016231	-2.344035	-1.457022
1	1.078128	-2.151529	0.844654
1	1.293006	-3.850532	0.372941
1	2.448521	-3.079368	1.473794
1	4.349592	-3.289343	-1.764771
1	4.264858	-3.910224	-0.108204
1	3.120162	-4.481949	-1.313969
6	-1.853157	-2.603994	-0.802983
6	-2.294940	-3.525398	-1.947075
6	-1.937929	-3.323635	0.546780
1	-0.805286	-2.342509	-0.968203
1	-2.208477	-3.023455	-2.913631
1	-1.677263	-4.426731	-1.969709
1	-3.336550	-3.832846	-1.819439
1	-1.600713	-2.674358	1.356695
1	-2.961361	-3.639761	0.765064
1	-1.309355	-4.217498	0.535368
6	-1.572930	0.136872	4.051885
6	-2.052475	0.154973	5.152863
1	-2.477616	0.171391	6.127482
35	-0.847081	0.106659	2.394017

IDipp2-ICCH

7	1.122772	0.047090	-1.418771
6	0.042285	0.035008	-0.596132
7	-0.996839	0.059719	-1.470833
6	-0.580462	0.080904	-2.793678
6	0.771799	0.083238	-2.760345
1	1.506600	0.104373	-3.547774
1	-1.275525	0.094971	-3.616562
6	2.473782	0.076268	-0.932776
6	3.017366	1.307093	-0.554527
6	3.179524	-1.132649	-0.846376
6	4.323225	1.307106	-0.059735
6	4.480269	-1.080074	-0.350343
6	5.045347	0.128254	0.042711
1	4.775924	2.242591	0.249887
1	5.060372	-1.990572	-0.264875
1	6.056918	0.147216	0.431267
6	-2.368821	-0.005557	-1.051096
6	-3.091283	1.190618	-0.934525
6	-2.914742	-1.259633	-0.761803
6	-4.411469	1.100716	-0.498646

6	-4.241270	-1.297187	-0.327654
6	-4.979670	-0.131479	-0.194435
1	-5.004954	2.000264	-0.391937
1	-4.698070	-2.251751	-0.091018
1	-6.006988	-0.179797	0.147429
6	2.524527	-2.451043	-1.225574
6	1.749414	-3.021731	-0.028685
6	3.520569	-3.484116	-1.757168
1	1.802983	-2.249151	-2.022506
1	0.984991	-2.325913	0.322904
1	1.263992	-3.961798	-0.305848
1	2.431958	-3.219731	0.802612
1	4.143744	-3.072073	-2.553997
1	4.176528	-3.852613	-0.964563
1	2.979574	-4.345764	-2.154078
6	-2.128631	-2.549406	-0.923697
6	-2.735614	-3.412165	-2.037076
6	-2.050079	-3.326238	0.394661
1	-1.106423	-2.297106	-1.215721
1	-2.768603	-2.869095	-2.984522
1	-2.143434	-4.319476	-2.179544
1	-3.756386	-3.712430	-1.785425
1	-1.606788	-2.714524	1.182641
1	-3.042279	-3.644100	0.726022
1	-1.437840	-4.222349	0.266570
6	-2.436415	2.532040	-1.222355
6	-1.708631	3.047980	0.027724
6	-3.423512	3.582592	-1.736010
1	-1.685794	2.376166	-2.002687
1	-0.947150	2.343360	0.367592
1	-1.226749	4.006764	-0.183833
1	-2.420554	3.193897	0.845026
1	-4.015420	3.206055	-2.573231
1	-4.109418	3.905828	-0.949013
1	-2.877523	4.467175	-2.070723
6	2.244520	2.609982	-0.666008
6	2.948075	3.583718	-1.618802
6	2.034259	3.242479	0.714078
1	1.257398	2.392180	-1.080891
1	3.085961	3.139648	-2.607371
1	2.357601	4.496450	-1.729690
1	3.932279	3.866750	-1.236327
1	1.512472	2.555363	1.383151
1	2.990636	3.505765	1.174220
1	1.441109	4.156021	0.624571
6	-0.250031	-0.156381	4.308743
6	-0.327352	-0.207104	5.510038
1	-0.395481	-0.251677	6.571081
53	-0.119983	-0.072226	2.281534

IAd2-ClCN

7	-0.000000	1.065701	-1.175877
6	0.000000	-0.000000	-0.322205
7	-0.000000	-1.065701	-1.175877
6	0.000000	-0.674103	-2.505753

6	0.000000	0.674103	-2.505753
1	-0.000000	1.365605	-3.330605
1	-0.000000	-1.365605	-3.330605
6	0.000000	-2.489396	-0.787834
6	0.000000	-2.631267	0.736248
6	1.256375	-3.176973	-1.352839
6	-1.256375	-3.176973	-1.352839
1	0.879935	-2.127625	1.144608
1	-0.879935	-2.127625	1.144608
6	0.000000	-4.117159	1.124722
1	2.143171	-2.672861	-0.954450
1	1.281653	-3.074611	-2.442807
6	1.255851	-4.664780	-0.971148
1	-2.143171	-2.672861	-0.954450
1	-1.281653	-3.074611	-2.442807
6	-1.255851	-4.664780	-0.971148
1	0.000000	-4.193897	2.215586
6	1.254008	-4.797835	0.558170
6	-1.254008	-4.797835	0.558170
1	2.150413	-5.139901	-1.383655
6	0.000000	-5.337693	-1.546322
1	-2.150413	-5.139901	-1.383655
1	1.268788	-5.855333	0.842283
1	2.153568	-4.333765	0.976025
1	-2.153568	-4.333765	0.976025
1	-1.268788	-5.855333	0.842283
1	0.000000	-5.260596	-2.639266
1	0.000000	-6.403434	-1.294955
6	0.000000	2.489396	-0.787834
6	1.256375	3.176973	-1.352839
6	0.000000	2.631267	0.736248
6	-1.256375	3.176973	-1.352839
1	2.143171	2.672861	-0.954450
1	1.281653	3.074611	-2.442807
6	1.255851	4.664780	-0.971148
1	0.879935	2.127625	1.144608
1	-0.879935	2.127625	1.144608
6	0.000000	4.117159	1.124722
1	-1.281653	3.074611	-2.442807
1	-2.143171	2.672861	-0.954450
6	-1.255851	4.664780	-0.971148
1	2.150413	5.139901	-1.383655
6	1.254008	4.797835	0.558170
6	0.000000	5.337693	-1.546322
1	0.000000	4.193897	2.215586
6	-1.254008	4.797835	0.558170
1	-2.150413	5.139901	-1.383655
1	1.268788	5.855333	0.842283
1	2.153568	4.333765	0.976025
1	0.000000	5.260596	-2.639266
1	0.000000	6.403434	-1.294955
1	-2.153568	4.333765	0.976025
1	-1.268788	5.855333	0.842283
6	-0.000000	0.000000	4.459511
17	-0.000000	0.000000	2.815479
7	-0.000000	0.000000	5.610376

IAd2-BrCN

7	-0.000000	1.066840	-1.372724
6	-0.000000	-0.000000	-0.518264
7	-0.000000	-1.066840	-1.372724
6	-0.000000	-0.673465	-2.701586
6	0.000000	0.673465	-2.701586
1	-0.000000	1.364375	-3.526573
1	-0.000000	-1.364375	-3.526573
6	-0.000000	-2.497794	-1.005360
6	0.000000	-2.669244	0.514289
6	1.256870	-3.174798	-1.582647
6	-1.256870	-3.174798	-1.582647
1	0.881083	-2.176210	0.932616
1	-0.881083	-2.176210	0.932616
6	0.000000	-4.161987	0.875793
1	2.143518	-2.678633	-1.174077
1	1.283770	-3.052852	-2.670368
6	1.255853	-4.669513	-1.229507
1	-2.143518	-2.678633	-1.174077
1	-1.283770	-3.052852	-2.670368
6	-1.255853	-4.669513	-1.229507
1	0.000000	-4.257221	1.965079
6	1.253977	-4.831970	0.296908
6	-1.253977	-4.831970	0.296908
1	2.150409	-5.136408	-1.651227
6	-0.000000	-5.330977	-1.817764
1	-2.150409	-5.136408	-1.651227
1	1.268182	-5.894616	0.560914
1	2.153574	-4.376409	0.723871
1	-2.153574	-4.376409	0.723871
1	-1.268182	-5.894616	0.560914
1	-0.000000	-5.232689	-2.909025
1	-0.000000	-6.401366	-1.587190
6	0.000000	2.497794	-1.005360
6	1.256870	3.174798	-1.582647
6	0.000000	2.669244	0.514289
6	-1.256870	3.174798	-1.582647
1	2.143518	2.678633	-1.174077
1	1.283770	3.052852	-2.670368
6	1.255853	4.669513	-1.229507
1	0.881083	2.176210	0.932616
1	-0.881083	2.176210	0.932616
6	0.000000	4.161987	0.875793
1	-1.283770	3.052852	-2.670368
1	-2.143518	2.678633	-1.174077
6	-1.255853	4.669513	-1.229507
1	2.150409	5.136408	-1.651227
6	1.253977	4.831970	0.296908
6	0.000000	5.330977	-1.817764
1	0.000000	4.257221	1.965079
6	-1.253977	4.831970	0.296908
1	-2.150409	5.136408	-1.651227
1	1.268182	5.894616	0.560914
1	2.153574	4.376409	0.723871
1	0.000000	5.232689	-2.909025

1	0.000000	6.401366	-1.587190
1	-2.153574	4.376409	0.723871
1	-1.268182	5.894616	0.560914
6	0.000000	0.000000	4.431914
35	0.000000	0.000000	2.624627
7	0.000000	0.000000	5.583463

IAd2-ICN

7	-0.000000	1.068832	-1.531864
6	0.000000	-0.000000	-0.676348
7	-0.000000	-1.068832	-1.531864
6	0.000000	-0.672546	-2.859094
6	0.000000	0.672546	-2.859094
1	-0.000000	1.362338	-3.684459
1	-0.000000	-1.362338	-3.684459
6	0.000000	-2.510643	-1.195164
6	0.000000	-2.725854	0.316955
6	1.257665	-3.171815	-1.789986
6	-1.257665	-3.171815	-1.789986
1	0.882699	-2.248832	0.749626
1	-0.882699	-2.248832	0.749626
6	0.000000	-4.227780	0.638637
1	2.144112	-2.687711	-1.366769
1	1.287119	-3.021513	-2.873780
6	1.255886	-4.675807	-1.478858
1	-2.144112	-2.687711	-1.366769
1	-1.287119	-3.021513	-2.873780
6	-1.255886	-4.675807	-1.478858
1	0.000000	-4.350070	1.725082
6	1.254033	-4.881406	0.042192
6	-1.254033	-4.881406	0.042192
1	2.150426	-5.130243	-1.913851
6	0.000000	-5.319934	-2.085919
1	-2.150426	-5.130243	-1.913851
1	1.267678	-5.950920	0.276379
1	2.153543	-4.438389	0.482228
1	-2.153543	-4.438389	0.482228
1	-1.267678	-5.950920	0.276379
1	0.000000	-5.190515	-3.173947
1	0.000000	-6.396423	-1.886182
6	0.000000	2.510643	-1.195164
6	1.257665	3.171815	-1.789986
6	0.000000	2.725854	0.316955
6	-1.257665	3.171815	-1.789986
1	2.144112	2.687711	-1.366769
1	1.287119	3.021513	-2.873780
6	1.255886	4.675807	-1.478858
1	0.882699	2.248832	0.749626
1	-0.882699	2.248832	0.749626
6	0.000000	4.227780	0.638637
1	-1.287119	3.021513	-2.873780
1	-2.144112	2.687711	-1.366769
6	-1.255886	4.675807	-1.478858
1	2.150426	5.130243	-1.913851

6	1.254033	4.881406	0.042192
6	0.000000	5.319934	-2.085919
1	0.000000	4.350070	1.725082
6	-1.254033	4.881406	0.042192
1	-2.150426	5.130243	-1.913851
1	1.267678	5.950920	0.276379
1	2.153543	4.438389	0.482228
1	0.000000	5.190515	-3.173947
1	0.000000	6.396423	-1.886182
1	-2.153543	4.438389	0.482228
1	-1.267678	5.950920	0.276379
6	0.000000	0.000000	4.501098
53	0.000000	0.000000	2.475464
7	0.000000	0.000000	5.653761

IAd2-ClCCH

7	-0.000000	1.064843	-1.225481
6	-0.000000	-0.000000	-0.371879
7	-0.000000	-1.064843	-1.225481
6	-0.000000	-0.674505	-2.556147
6	0.000000	0.674505	-2.556147
1	-0.000000	1.366392	-3.380943
1	-0.000000	-1.366392	-3.380943
6	-0.000000	-2.483633	-0.824159
6	0.000000	-2.605640	0.702247
6	1.255971	-3.177907	-1.381319
6	-1.255971	-3.177907	-1.381319
1	0.877629	-2.094430	1.105651
1	-0.877629	-2.094430	1.105651
6	0.000000	-4.086464	1.108340
1	2.142607	-2.668625	-0.989312
1	1.280504	-3.088755	-2.472673
6	1.255865	-4.660880	-0.980837
1	-2.142607	-2.668625	-0.989312
1	-1.280504	-3.088755	-2.472673
6	-1.255865	-4.660880	-0.980837
1	0.000000	-4.149437	2.200124
6	1.253818	-4.774619	0.550164
6	-1.253818	-4.774619	0.550164
1	2.150403	-5.141500	-1.387316
6	-0.000000	-5.341169	-1.547331
1	-2.150403	-5.141500	-1.387316
1	1.269022	-5.828766	0.847156
1	2.153173	-4.304750	0.961919
1	-2.153173	-4.304750	0.961919
1	-1.269022	-5.828766	0.847156
1	-0.000000	-5.277860	-2.641223
1	-0.000000	-6.403737	-1.282372
6	0.000000	2.483633	-0.824159
6	1.255971	3.177907	-1.381319
6	0.000000	2.605640	0.702247
6	-1.255971	3.177907	-1.381319
1	2.142607	2.668625	-0.989312
1	1.280504	3.088755	-2.472673

6	1.255865	4.660880	-0.980837
1	0.877629	2.094430	1.105651
1	-0.877629	2.094430	1.105651
6	0.000000	4.086464	1.108340
1	-1.280504	3.088755	-2.472673
1	-2.142607	2.668625	-0.989312
6	-1.255865	4.660880	-0.980837
1	2.150403	5.141500	-1.387316
6	1.253818	4.774619	0.550164
6	0.000000	5.341169	-1.547331
1	0.000000	4.149437	2.200124
6	-1.253818	4.774619	0.550164
1	-2.150403	5.141500	-1.387316
1	1.269022	5.828766	0.847156
1	2.153173	4.304750	0.961919
1	0.000000	5.277860	-2.641223
1	0.000000	6.403737	-1.282372
1	-2.153173	4.304750	0.961919
1	-1.269022	5.828766	0.847156
6	0.000000	0.000000	4.559293
6	0.000000	0.000000	5.758254
1	0.000000	0.000000	6.821166
17	0.000000	0.000000	2.911830

IAd2-BrCCH

7	-0.000000	1.065509	-1.447669
6	0.000000	-0.000000	-0.593164
7	-0.000000	-1.065509	-1.447669
6	0.000000	-0.674076	-2.777643
6	0.000000	0.674076	-2.777643
1	-0.000000	1.365751	-3.602383
1	-0.000000	-1.365751	-3.602383
6	0.000000	-2.489275	-1.060437
6	0.000000	-2.630340	0.463119
6	1.256268	-3.176857	-1.625625
6	-1.256268	-3.176857	-1.625625
1	0.878713	-2.125890	0.872512
1	-0.878713	-2.125890	0.872512
6	0.000000	-4.115518	0.852614
1	2.142854	-2.672492	-1.227185
1	1.281662	-3.075233	-2.715748
6	1.255864	-4.664517	-1.243031
1	-2.142854	-2.672492	-1.227185
1	-1.281662	-3.075233	-2.715748
6	-1.255864	-4.664517	-1.243031
1	0.000000	-4.189939	1.943603
6	1.253827	-4.796915	0.286447
6	-1.253827	-4.796915	0.286447
1	2.150404	-5.140012	-1.655415
6	0.000000	-5.337742	-1.817879
1	-2.150404	-5.140012	-1.655415
1	1.268717	-5.854534	0.570675
1	2.153201	-4.332366	0.704101
1	-2.153201	-4.332366	0.704101

1	-1.268717	-5.854534	0.570675
1	0.000000	-5.261109	-2.910924
1	0.000000	-6.403431	-1.565899
6	0.000000	2.489275	-1.060437
6	1.256268	3.176857	-1.625625
6	0.000000	2.630340	0.463119
6	-1.256268	3.176857	-1.625625
1	2.142854	2.672492	-1.227185
1	1.281662	3.075233	-2.715748
6	1.255864	4.664517	-1.243031
1	0.878713	2.125890	0.872512
1	-0.878713	2.125890	0.872512
6	0.000000	4.115518	0.852614
1	-1.281662	3.075233	-2.715748
1	-2.142854	2.672492	-1.227185
6	-1.255864	4.664517	-1.243031
1	2.150404	5.140012	-1.655415
6	1.253827	4.796915	0.286447
6	0.000000	5.337742	-1.817879
1	0.000000	4.189939	1.943603
6	-1.253827	4.796915	0.286447
1	-2.150404	5.140012	-1.655415
1	1.268717	5.854534	0.570675
1	2.153201	4.332366	0.704101
1	0.000000	5.261109	-2.910924
1	0.000000	6.403431	-1.565899
1	-2.153201	4.332366	0.704101
1	-1.268717	5.854534	0.570675
6	-0.000000	0.000000	4.532838
6	-0.000000	0.000000	5.733416
1	-0.000000	0.000000	6.796922
35	-0.000000	0.000000	2.730374

IAd2-ICCH

7	0.000000	1.066599	-1.637145
6	0.000000	0.000000	-0.781247
7	-0.000000	-1.066599	-1.637145
6	-0.000000	-0.673398	-2.966054
6	0.000000	0.673398	-2.966054
1	0.000000	1.364416	-3.790970
1	-0.000000	-1.364416	-3.790970
6	-0.000000	-2.497930	-1.271830
6	-0.000000	-2.669563	0.246941
6	1.256795	-3.174766	-1.849503
6	-1.256795	-3.174766	-1.849503
1	0.880298	-2.176009	0.666029
1	-0.880298	-2.176009	0.666029
6	-0.000000	-4.161523	0.609418
1	2.143275	-2.678453	-1.440827
1	1.283767	-3.053248	-2.937340
6	1.255885	-4.669454	-1.495866
1	-2.143275	-2.678453	-1.440827
1	-1.283767	-3.053248	-2.937340
6	-1.255885	-4.669454	-1.495866

1	-0.000000	-4.254645	1.698909
6	1.253917	-4.831784	0.030671
6	-1.253917	-4.831784	0.030671
1	2.150404	-5.136600	-1.917611
6	-0.000000	-5.331069	-2.083960
1	-2.150404	-5.136600	-1.917611
1	1.268447	-5.894601	0.294473
1	2.153237	-4.375707	0.457614
1	-2.153237	-4.375707	0.457614
1	-1.268447	-5.894601	0.294473
1	-0.000000	-5.232910	-3.175289
1	-0.000000	-6.401472	-1.853086
6	0.000000	2.497930	-1.271830
6	1.256795	3.174766	-1.849503
6	0.000000	2.669563	0.246941
6	-1.256795	3.174766	-1.849503
1	2.143275	2.678453	-1.440827
1	1.283767	3.053248	-2.937340
6	1.255885	4.669454	-1.495866
1	0.880298	2.176009	0.666029
1	-0.880298	2.176009	0.666029
6	0.000000	4.161523	0.609418
1	-1.283767	3.053248	-2.937340
1	-2.143275	2.678453	-1.440827
6	-1.255885	4.669454	-1.495866
1	2.150404	5.136600	-1.917611
6	1.253917	4.831784	0.030671
6	0.000000	5.331069	-2.083960
1	0.000000	4.254645	1.698909
6	-1.253917	4.831784	0.030671
1	-2.150404	5.136600	-1.917611
1	1.268447	5.894601	0.294473
1	2.153237	4.375707	0.457614
1	0.000000	5.232910	-3.175289
1	0.000000	6.401472	-1.853086
1	-2.153237	4.375707	0.457614
1	-1.268447	5.894601	0.294473
6	-0.000000	-0.000000	4.594785
6	-0.000000	-0.000000	5.798155
1	-0.000000	-0.000000	6.862263
53	-0.000000	-0.000000	2.590146
